# Histone deacetylase inhibitor panobinostat induces antitumor activity in epithelioid sarcoma and rhabdoid tumor by growth factor receptor modulation

**DOI:** 10.1186/s12885-021-08579-w

**Published:** 2021-07-20

**Authors:** Anne Catherine Harttrampf, Maria Eugenia Marques da Costa, Aline Renoult, Estelle Daudigeos-Dubus, Birgit Geoerger

**Affiliations:** 1 Gustave Roussy Cancer Center, INSERM U1015, Université Paris-Saclay, Villejuif, France; 2 Department of Pediatric and Adolescent Oncology, Gustave Roussy Cancer Center, 114 Rue Edouard Vaillant, 94805 Villejuif, France; 3 Present address: Institute of Research in Immunology and Cancer, Dr Trang Hoang Laboratory, Université de Montréal, Montreal, Québec Canada; 4 Present address: AP-HP Nord, DMU Neurosciences, Service de Neurologie, FHU NeuroVasc, Université de Paris, Paris, France

**Keywords:** Epithelioid sarcoma, Rhabdoid tumor, HDAC inhibition, Epithelial-to mesenchymal transition, Growth factor receptors

## Abstract

**Background:**

Epithelioid sarcomas and rhabdoid tumors are rare, aggressive malignancies with poor prognosis. Both are characterized by INI1 alterations and deregulation of growth factor receptors albeit their interaction has not been elucidated.

**Methods:**

In this study, we investigated the activity of a panel of epigenetic modulators and receptor tyrosine kinase inhibitors in vitro on respective cell lines as well as on primary patient-derived epithelioid sarcoma cells, and in vivo on xenografted mice. Focusing on histone deacetylase (HDAC) inhibitors, we studied the mechanism of action of this class of agents, its effect on growth factor receptor regulation, and changes in epithelial-to-mesenchymal transition by using cell- and RT-qPCR-based assays.

**Results:**

Pan-HDAC inhibitor panobinostat exhibited potent anti-proliferative activity at low nanomolar concentrations in A204 rhabdoid tumor, and VAESBJ/GRU1 epithelioid sarcoma cell lines, strongly induced apoptosis, and resulted in significant tumor growth inhibition in VAESBJ xenografts. It differentially regulated EGFR, FGFR1 and FGFR2, leading to downregulation of EGFR in epithelioid sarcoma and to mesenchymal-to-epithelial transition whereas in rhabdoid tumor cells, EGFR was strongly upregulated and reinforced the mesenchymal phenotype. All three cell lines were rendered more susceptible towards combination with EGFF inhibitor erlotinib, further enhancing apoptosis.

**Conclusions:**

HDAC inhibitors exhibit significant anticancer activity due to their multifaceted actions on cytotoxicity, differentiation and drug sensitization. Our data suggest that the tailored, tissue-specific combination of HDAC inhibitors with therapeutics which target cellular salvage mechanisms might increase their therapeutic relevance.

**Supplementary Information:**

The online version contains supplementary material available at 10.1186/s12885-021-08579-w.

## Background

Epithelioid sarcomas and rhabdoid tumors are rare, highly aggressive malignancies with poor prognosis. Epithelioid sarcoma affects adolescents and adults, and peculiarly displays both mesenchymal and epithelial features [1, 2]. The classical form affects younger patients and is mainly localized to the distal upper extremities, the proximal type involves the trunk and is characterized by a more aggressive clinical course. Cornerstone of treatment is exhaustive surgery but a strong tendency to local recurrence, lymphatic and eventually pulmonary spread occurring in 30–50%, and chemoresistance, result in dismal outcome [3]. The proximal type is histologically different including rhabdoid features, but it is not clear whether this alone accounts for its more aggressive clinical course, as surgical resectability might be impaired due to deeper infiltration and anatomic location [4]. Rhabdoid tumors predominantly occur in children < 3 years affecting brain (atypical teratoid rhabdoid tumor, (ATRT)) and kidney but can occur virtually in any anatomic location; 6-year overall and event-free survival is around 45% with maximally intensive treatments [5].

Shared hallmark finding of both malignancies is inactivation of *SMARCB1* by mutation and/or deletion [6, 7]. INI1, core component of the SWI/SNF chromatin remodeling complex whose role as bona fide tumor suppressor has been validated, regulates gene expression of several genes by acting on chromatin conformation in an ATP-dependent manner [8, 9]. Correspondent loss of INI1 expression is present in rhabdoid tumor and 80–90% of epithelioid sarcoma [1, 7]. INI1 inactivation deregulates multiple axes maintaining physiologic cell regulation promoting dedifferentiation, tumor cell growth, and progression, which led to the clinical evaluation of histone methyltransferase Enhancer of zeste homolog 2 (EZH2) inhibitor tazemetostat in INI-negative tumors (NCT02601937). EZH2 inhibition alone or in combination with histone deacetylases (HDAC) inhibitors has been suggested for both tumor types, but antagonistic effects of this drug combination on in vitro rhabdoid tumor cell proliferation have also been demonstrated [10–12]. HDAC inhibitors influence several cellular regulatory processes by histone acetylation, leading to a transcription-permissive chromatin state, and by acetylation of non-histone proteins which induce apoptosis and cellular differentiation in cancer cells [13]. Inhibition of histone deacetylases was reported to compensate for the loss of INI1 by restoring tumor suppressor activity in rhabdoid tumor [14]. Low-dose pan-HDAC inhibition induced multilinear differentiation in rhabdoid tumor cells and led to tumor growth inhibition in vivo [15]. Antitumor activity by HDAC inhibition was also shown in epithelioid sarcoma, and downregulation of EZH2 was linked to inhibition of HDACs 1 and 2 [10]. Both HDACs and the EZH2-containing PRC2 complex have been linked to the transcriptional repression of E-Cadherin via its regulator Snail, which is a key event in epithelial-to-mesenchymal transition (EMT) [16, 17]. EMT is a cellular plasticity process elementary to both physiological embryonic development, and cancer progression and invasion which is largely steered on the epigenetic level. Epithelial differentiation of tumor cells is linked to increased responsiveness to targeted agents and chemotherapeutics [18].

In both tumors aberrantly activated growth factor receptors have been identified albeit their relation to INI1 inactivation is unclear. They include Fibroblast Growth Factor Receptors 1, − 2 (FGFR1, FGFR2), Platelet-Derived Growth Factor Receptor A (PDGFRA), and Epidermal Growth Factor Receptor (EGFR) in rhabdoid tumor, and evidence has been provided that their regulation, at least for certain receptors, requires intact INI1 function [19–22]. In epithelioid sarcoma, EGFR overexpression is characteristic and combined inhibition of EGFR and mTOR has been suggested [23, 24].

(Epi-)genetic parallels in epithelioid sarcoma and rhabdoid tumor, associated with growth factor receptor deregulation, prompted us to take further insight into the effects of epigenetic modulation and interplay between growth factor receptor expression and signaling. We show that both cancers display high sensitivity towards pan-HDAC inhibitor panobinostat by proliferation inhibition and apoptosis induction. A second important property of HDAC inhibitors is if to modify or change the expression of proteins which can serve as drug targets themselves. We show that differential regulation of *EGFR* and *FGFR2*, and partial reversion of EMT in epithelioid sarcoma is induced by HDAC inhibitor panobinostat. Panobinostat increases sensitivity to EGFR inhibition, suggesting the combination of epigenetic compounds with specific inhibitors of emerging targets for the treatment of these malignancies.

## Methods

### Cell lines and cell culture

Rhabdoid tumor cell line A204 was purchased from Leibniz Institute DSMZ (German Collection of Microrganisms and Cell Cultures), epithelioid sarcoma cell line VAESBJ from ATCC® (American Type Culture Collection). Epithelioid sarcoma cell line GRU-1 was kindly provided by C. Mahotka (University of Düsseldorf, Germany). MOSC-GR-001-ES primary cells were derived from a thoracic biopsy of a patient with relapsed epithelioid sarcoma, established in our laboratory. Cells were maintained in Dulbecco‘s Modified Eagle Medium (DMEM) GlutaMAX™ (Gibco, Life Technologies, Villebon-sur-Yvette, France), containing 10% FBS (Fetal bovine serum; Sigma-Aldrich, St. Quentin Fallavier, France), and 1% Minimal Essential Medium Non-Essential Amino Acids (Gibco, Life Technologies) for epithelioid sarcoma cells, at 37 °C in a 5% CO_2_ atmosphere. Presence of mycoplasma was regularly excluded.

### Reagents

Erlotinib, pazopanib, everolimus, mocetinostat, vorinostat, panobinostat were purchased (LC Laboratories, Woburn, USA). Regorafenib (BAY 73–4506) was provided by Bayer Pharma AG Germany, EPZ011898–9 by Epizyme (Cambridge, MA, USA). Compounds were dissolved in dimethylsulfoxide (DMSO) and stored in 10 mmol/L stock solutions. Recombinant hEGF (Cell Signaling, Saint Quentin Yvelines, France), recombinant hFGF basic, and recombinant hKGF/FGF7 (R&D Systems, Lille, France) were dissolved in PBS.

### Proliferation assays

Cells were seeded in 96-well plates at 5000 (A204 and VAESBJ), 10–15,000 (GRU-1), or 1000 cells per well (MOSC-GR-001-ES); for EPZ-011989-8 experiments at 100 (VAESBJ, A204) and 500 cells per well (GRU1). Drug dilutions were administered in increasing concentrations up to 10 μM. Longitudinal live cell imaging by taking phase contrast pictures every 2–4 h and calculation of cell confluence per well was carried out using the IncuCyte ZOOM® and software (Essen BioScience Ltd., Hertfordshire, UK). Selected drug or drug combination cell viability tests were determined using the CellTiter 96® AQueous One Solution Cell Proliferation Assay (MTS; Promega, Charbonnières-les-Bains, France). Cell confluence or viability quantified by measuring the optical densitometry by spectrophotometry at 490 nm was determined after 72 h except for MOSC-GR-001-ES (144 h), compared to vehicle controls (DMSO). All tests were performed in a minimum of quadruplicate, repeated at least twice.

### Apoptosis assays

Cells were seeded in 6-well-plates in triplicates for 24, 48 and 72 h with panobinostat at 10, 50 and 100 nM for A204, and 25, 125 and 250 nM for VAESBJ and GRU1 (corresponding to IC_50_, 5x IC_50_ and 10x IC_50_), determined previously by IncuCyte ZOOM® versus empty vehicle. Cells were stained with FITC Annexin V and propidium iodide (FITC Annexin V Apoptosis Detection Kit I, BD Biosciences, Le Pont de Claix, France), and analyzed by Fluorescence-activated cell-sorting (FACS), using the BD Accuri C6 (BD Biosciences).

### Migration and invasion assays

Migration and invasion inhibition were analyzed by wound scratch method using the IncuCyte® WoundMaker™ (Essen BioScience Ltd). Cells were seeded in appropriate 96-well ImageLock tissue culture plates coated with 0.1 mg/mL BD Matrigel™ (Corning, Boulogne-Billancourt, France) at 20000, 25000 and 30,000 cells/well for migration, and 30,000, 35,000 and 45,000 cells/well for invasion for VAESBJ, A204 and GRU1, respectively. Cells were starved using minimal essential medium (MEM; Gibco, Life Technologies) for 4–6 h, before applying the wound scratch, and addition of drug concentrations and/or growth factors. For invasion assays, BD Matrigel™ (6 mg/mL) was applied to each well before treatment. Results were analyzed every 2–4 h using IncuCyte ZOOM®. All tests were performed in quadruplicates, independently repeated at least twice.

### Reverse transcription quantitative real-time PCR (RT-qPCR)

Total RNA extracted using RNeasy Mini kit (Qiagen, Courtaboeuf, France) was reverse transcribed with M-MLV RT buffer pack (Life Technologies), and real-time PCR (qPCR) was performed on StepOnePlus PCR System (AB Applied Biosystems, Villebon-sur-Yvette, France) with SYBR Green (Thermo Scientific). GAPDH expression served as internal control to normalize expression using the2^-ΔΔCt^ method. Each experiment was carried out in triplicate at least three times independently. Primer sequences used for amplification are available upon request.

### Western blot analysis

Cell pellets were lyzed using TNEN buffer 5 mM (50 mM TrisHcl, 250 mM sodium chloride, 5 mM EDTA pH 8, 1% NP_4_O) and cocktails of protease and phosphatase inhibitors. Equal amounts of protein (30 μg) were resolved by 4–15% precast SDS polyacrylamide gels and transferred onto PVDF membranes by the Trans-Blot® turbo™ Transfer Starter System (all Bio-Rad Laboratories, Marne-la-Coquette, France). Protein expression was detected using the primary antibodies p-ERK (Thr202/Tyr204), p-AKT (Ser473), p-SHC (Tyr239/240), p-GAB1 (Tyr627), AKT, ERK, STAT, EGFR, FGFR2, PARP, CASPASE 3, E-CADHERIN, SNAIL, SMARCB1, β-Actin HRP conjugate (1:1000 except p-AKT 1:250–1:500; all Cell Signaling), Acteyl-H4 (1:1000; Merck Millipore, Guyancourt, France), revealed by peroxidase-conjugated secondary anti-rabbit or anti-mouse antibodies (1:5000, Cell signaling), and ChemiDoc™ MP Imaging System (Clarity™ Western ECL Substrate, Bio-Rad Laboratories, or SuperSignal® WestFemto Maximum Sensitivity Substrate, Thermo Fisher Scientific, Villebon-sur-Yvette, France). Screening of phosphorylated receptor tyrosine kinases was performed using Human Phospho-RTK Array (R&D Systems).

All methods were carried out in accordance with relevant guidelines and regulations. Animal experiments were carried out under conditions established by the European Community (Directive 2010/63/UE) and in concordance with the ARRIVE guidelines. All experimental protocols were approved by the CEEA26 Ethics Committee and French Ministry of Research (MENESR, Ministère de l’éducation nationale, de l’enseignement supérieur et de la recherche; reference APAFIS #00328.01 and #9319-2017032011088915v3). Informed consent was obtained from the subject and from the parents.

### Experimental in vivo design

Antitumor activity was evaluated against VAESBJ and A204 xenografts in female Swiss athymic mice of 6–9 weeks of age (Charles River, Saint Germain Nuelles, France), established by subcutaneous injection of 5 × 10^6^ cells into both flanks. Animals bearing tumors of 80–300 mm^3^ were randomized into groups of six (regorafenib experiment) and eight animals (panobinostat experiment), and treated with sonificated panobinostat 8 or 12 mg/kg, or 5% glucose intraperitoneally three times a week [25]. Regorafenib was administered orally by gavage at 30 mg/kg for a minimum of 20 days [26]. Animals were followed daily for clinical status and three times per week for tumor growth and body weight. Antitumor activity was determined as described previously [26]. For pharmacodynamics analysis, primary tumors were harvested 24 h after a single dose of panobinostat, and total tumor lysates were generated as previously described [26].

### Statistical analysis

Statistical analyses were carried out with GraphPad Prism (v5.01). Fold-changes with indication of geometric mean, or percentage of transcript downregulation indicated in the text, were calculated according to the 2^-ΔΔCT^ method.

## Results

### Panobinostat exhibits strong antitumor activity in vitro

**Fig. 1:**
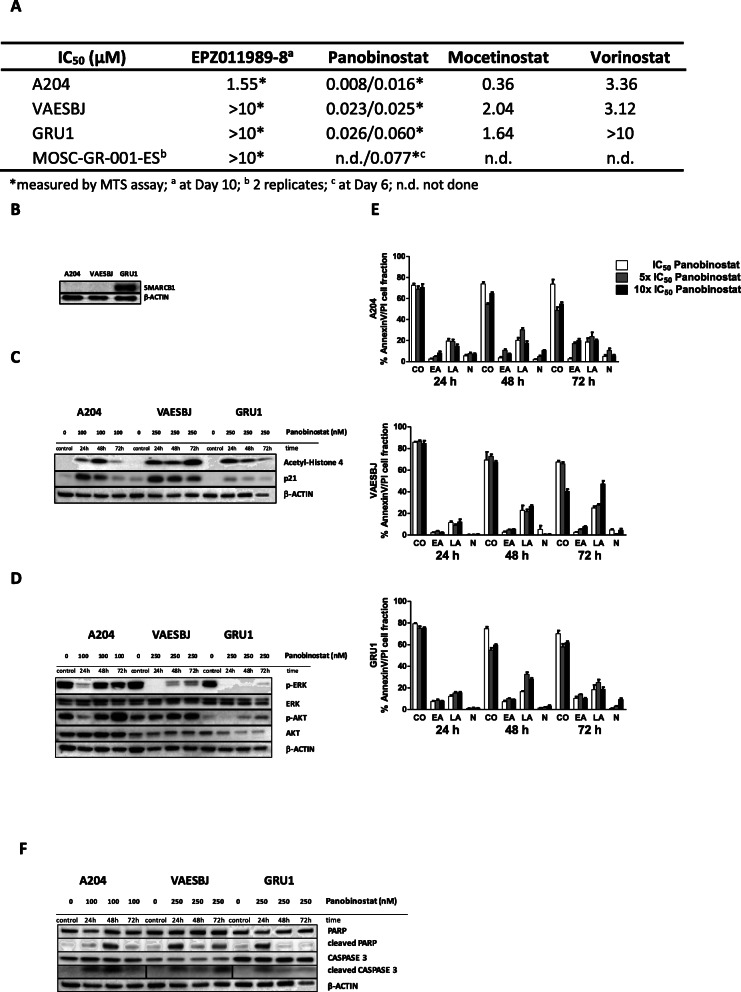
In vitro toxicity of epigenetic modulators is mediated through induction of apoptosis in epithelioid sarcoma and rhabdoid tumor cell lines. **A** Half-maximal inhibitory concentration (IC_50_) of epigenetic agents after 72 h, based on confluence (IncuCyte®) or measured by MTS assay (*), determined at Day 10^a^ or at Day 6^c^, in *N* ≥ 3 biological replicates (2 replicates^b^); n.d. = not done. Western Blot analyses showing: **B** INI1 expression in A204, VAESBJ and GRU1, with maintained expression in GRU1 epithelioid sarcoma; **C** Biological activity of panobinostat (10xIC_50_: 100 nM for A204, 250 nM for VAESBJ, GRU1) by induction of p21 and H4-acetylation; **D** Reduced ERK and AKT activity after 24, 48 and 72 h. **E** Induced apoptosis in a dose- and time-dependent manner measured by Annexin V- and PI-FACS analysis; graph presents means ± SEM of *N* = 3 replicates for 10/25 nM (IC_50_), 50/125 nM (5xIC_50_) or 100/250 nM (10xIC_50_) for rhabdoid tumor/epithelioid sarcoma cell lines as compared to control (CO), and percentage of cells in early apoptosis (EA), late apoptosis (LA), or necrosis (N). **F** Confirmation of apoptosis by the presence of cleaved PARP and cleaved Caspase 3 in Western Blot

The anti-proliferative effect of EZH2 inhibitor EPZ011989–8 and pan-HDAC inhibitors vorinostat, panobinostat (hydroxamates), and mocetinostat (benzamide) were screened using live cell imaging (IncuCyte ZOOM®) and/or MTS in VAESBJ and GRU1 epithelioid sarcoma and A204 rhabdoid tumor cell lines (Fig. 1A). Overall, panobinostat induced the strongest anti-proliferative activity with half-maximal inhibitory concentrations (IC_50_) between 8 and 26 nM determined by live cell imaging, and 16 and 60 nM by MTS at 72 h. IC_50_s of mocetinostat and vorinostat were considerably higher or not determinable. The most sensitive cell line, A204, also displayed intermediate sensitivity towards EPZ011989–8 whereas both epithelioid sarcoma cell lines were resistant. The sensitivity of the two epithelioid cell lines to panobinostat was comparable despite the fact that GRU1 has retained INI1 protein expression (Fig. 1B). We further tested panobinostat and EPZ011989–8 on primary patient-derived cells (MOS-GR-001-ES) of an epithelioid sarcoma in a 17-year-old patient. This specimen exhibits a heterozygous 8 Megabase deletion at 22q, involving *SMARCB1*, and has lost INI1 expression [27]. Similar to the cell lines, panobinostat IC_50_ settled with 77 nM in the 2-digit nanomolar range and cells were resistant to EZH2 inhibition (Fig. 1A). We confirmed biological activity of panobinostat by increased protein acetylation and induction of cell-cycle inhibitor p21 as biomarkers for successful HDAC inhibition (Fig. 1C). Panobinostat strongly reduced ERK phosphorylation in A204, VAESBJ and GRU1 cells, which was durable in epithelioid sarcoma cells (Fig. 1D). Inhibition of p-AKT was present in A204 and GRU1. Annexin V/propidium iodide-FACS analysis demonstrated strong induction of apoptosis in all three cell lines tested whereas necrosis was less prominent (Fig. 1E). Cleaved PARP and CASPASE 3 corroborated these findings (Fig. 1F). Taken together, panobinostat uniformly demonstrated strongest anti-proliferative activity by cell growth inhibition, and induced apoptosis-mediated cell death and was therefore selected for further functional evaluations.

### Rhabdoid tumor cells exhibit sensitivity towards multi-tyrosine kinase inhibition whereas oncogenic signaling is activated in epithelioid sarcoma

**Fig. 2:**
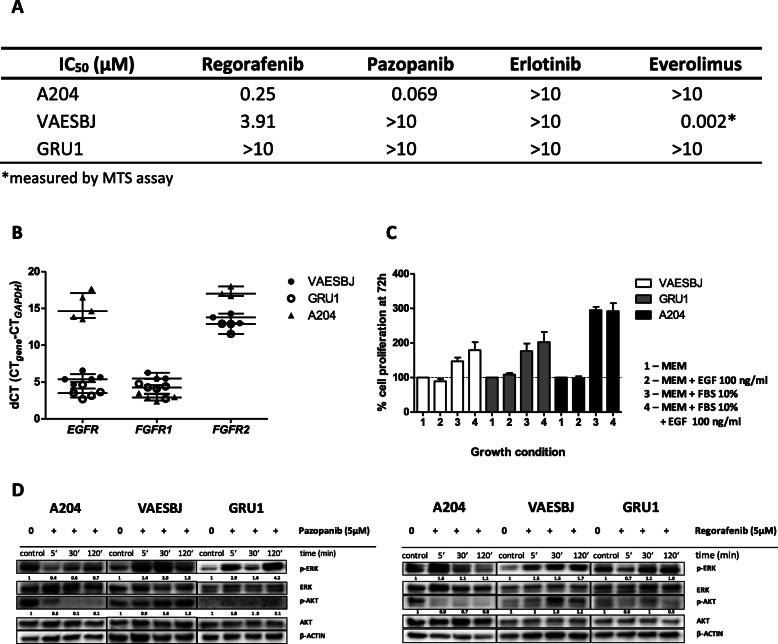
Rhabdoid tumor is more sensitive to in vitro inhibition of growth factor receptors than epithelioid sarcoma. **A** Half-maximal inhibitory concentration (IC_50_) after 72 h, based on confluence (IncuCyte®) or MTS assay (*) in *N* ≥ 3 biological replicates. **B** *EGFR*, *FGFR1* and FGFR2 mRNA expression levels of A204, VAESBJ, and GRU1 cells were determined by RT-qPCR. The scatter plot shows measurements of N ≥ 3 biological replicates in 3 technical replicates, normalized on *GAPDH* expression (dCT (cycle threshold) values; median ± IQR ). **C** Cell proliferation in response to EGF and FBS10% in serum-starved cells by IncuCyte®, graph represents means ± SEM of N ≥ 3 biological replicates. **D** Effects on AKT and ERK activity after treatment with pazopanib (left panel) and regorafenib (right panel) after 5, 30 and 120 min, assessed by Western blot (ratio candidate protein/β-ACTIN, related to expression of control)

Based on the overexpression of growth factor receptors in both tumor types, we selected multi-tyrosine kinase inhibitors (TKI), comprising FGFR which were either approved for the treatment of advanced soft tissue sarcoma (pazopanib) or in clinical investigation (regorafenib, NCT02085148), and erlotinib for EGFR inhibition. mTOR inhibitor everolimus was also evaluated. A204 proved to be the most sensitive cell line towards multi-tyrosine kinase inhibition of cell proliferation, but was resistant to erlotinib and everolimus (Fig. 2A). VAESBJ exhibited low sensitivity to regorafenib and resistance to pazopanib, but it was highly sensitive to everolimus; it was the only cell line in which panobinostat did not succeed to impair AKT phosphorylation (see also Fig. 1D). GRU1 was resistant to all agents tested. Despite *EGFR* overexpression, as confirmed by RT-qPCR (Fig. 2B), both epithelioid sarcoma cell lines were not sensitive to erlotinib. Cell proliferation was also not enhanced by specific stimulation with EGF compared to 10% FBS (Fig. 2C). In untreated cell lines, *FGFR1* expression was homogeneously strong across all three cell lines whereas *FGFR2* was low or hardly expressed (Fig. 2B). Cells were treated with pazopanib or regorafenib to evaluate and compare effects on intracellular signaling. A204 cells showed significant reduction of ERK and AKT phosphorylation in accordance with results from cell proliferation tests, while their increased phosphorylation was observed in both epithelioid sarcoma lines (Fig. 2D). In a phospho-RTK array using untreated VAESBJ cells, strongest phosphorylation was seen in EGFR; none of the other phosphorylated kinases EPHB1, − 2, − 6, RYK, or AXL, is a known target of regorafenib or pazopanib (data not shown). This suggests that blockade of tyrosine kinases comprised in the target spectrum of the respective drug results in increased activation of either non-inhibited tyrosine kinases, or alternative cellular responses leading to activation of MAPK- and AKT/mTOR signaling. To resume, agents targeting multiple receptor tyrosine kinases including FGFR showed in vitro sensitivity in rhabdoid tumor cells but activation of oncogenic intracellular signaling in epithelioid sarcoma.

### Panobinostat and regorafenib inhibit tumor growth in VAESBJ and A204 xenografts in vivo

**Fig. 3:**
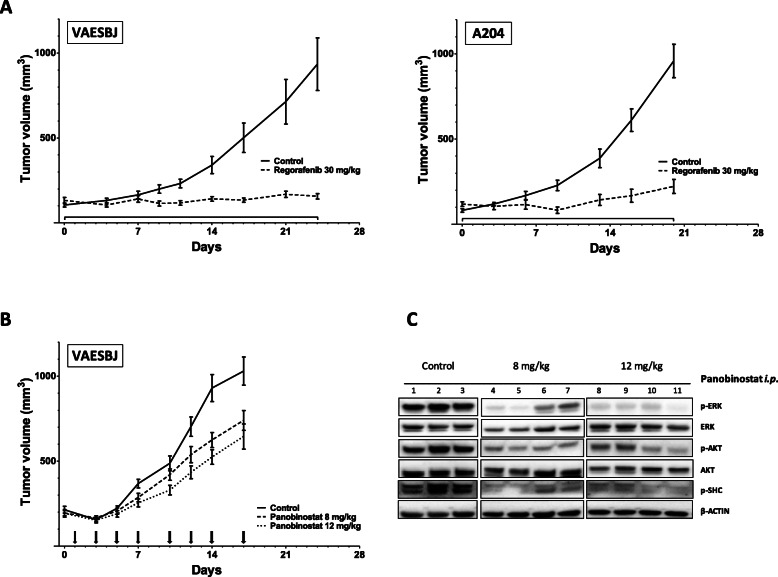
Regorafenib and panobinostat induce tumor growth inhibition in vivo*.* Mice xenografted with **A**, VAESBJ tumors and A204 tumors were treated daily with 30 mg/kg regorafenib *p.o.*; graph represents means ± SEM of *N* = 8 to 9 tumors per group. Statistical analysis in both experiments was performed using the nonparametric Mann-Whitney test; *p* < 0.001 each. **B** Animals bearing VAESBJ tumors were treated with 8 and 12 mg/kg panobinostat *i.p.* three times per week; graph represents means ± SEM of *N* = 11 to 16 tumors per group. Statistical analysis was performed using the nonparametric Kruskal-Wallis test; *p* < 0.05 for 8 mg/kg and *p* < 0.0005 for 12 mg/kg. **C** ERK and AKT activation in tumors harvested after 24 h of a single dose of 8 and 12 mg/kg panobinostat *i.p.* are shown by Western blot

Antitumor activity of panobinostat and regorafenib in vivo was explored in subcutaneous VAESBJ and A204 xenografts (Fig. 3). Regorafenib at 30 mg/kg resulted in significant VAESBJ tumor growth delay (TGD) of > 20.4 days and 97% tumor growth inhibition (TGI) at Day 24 (both < 0.0001; Mann-Whitney) compared to vehicle controls (Fig. 3A). In A204, regorafenib induced significant TGD of 13 days and TGI of 88% at Day 20 (both *p* < 0.001, Mann-Whitney). In addition, one partial and one complete response were observed in this model consistent with the cytotoxic sensitivity in vitro. No treatment-related toxicity was observed in the vehicle and the treatment groups in both models (data not shown). Furthermore, VAESBJ xenografts were treated with panobinostat intraperitoneally three times a week (Fig. 3B). This resulted in TGI at 8 mg/kg and 12 mg/kg with a mean tumor volume of 624 mm^3^ (*p* < 0.05) and 524 mm^3^ (*p* < 0.0005; Kruskal-Wallis test), respectively, as compared to controls (930 mm^3^) on Day 14. No treatment-related toxicity was observed in the vehicle and the 8 mg/kg treatment groups, whereas animals experienced a 3 to 16% weight loss on day 7 after five doses at 12 mg/kg, and 6 out of 8 mice were not treated at day 10 to recover. The experiment was stopped after 17 days due to ulceration of the model. Pharmacodynamics analysis showed strong reduction of intracellular growth-factor receptor-related signaling pathways (Fig. 3C). These data demonstrate that single-agent panobinostat and regorafenib possess significant in vivo antitumor activity.

### The differential regulation of EGFR induces mesenchymal-to-epithelial transition in epithelioid sarcoma but reinforces the mesenchymal phenotype of rhabdoid tumor

**Fig. 4:**
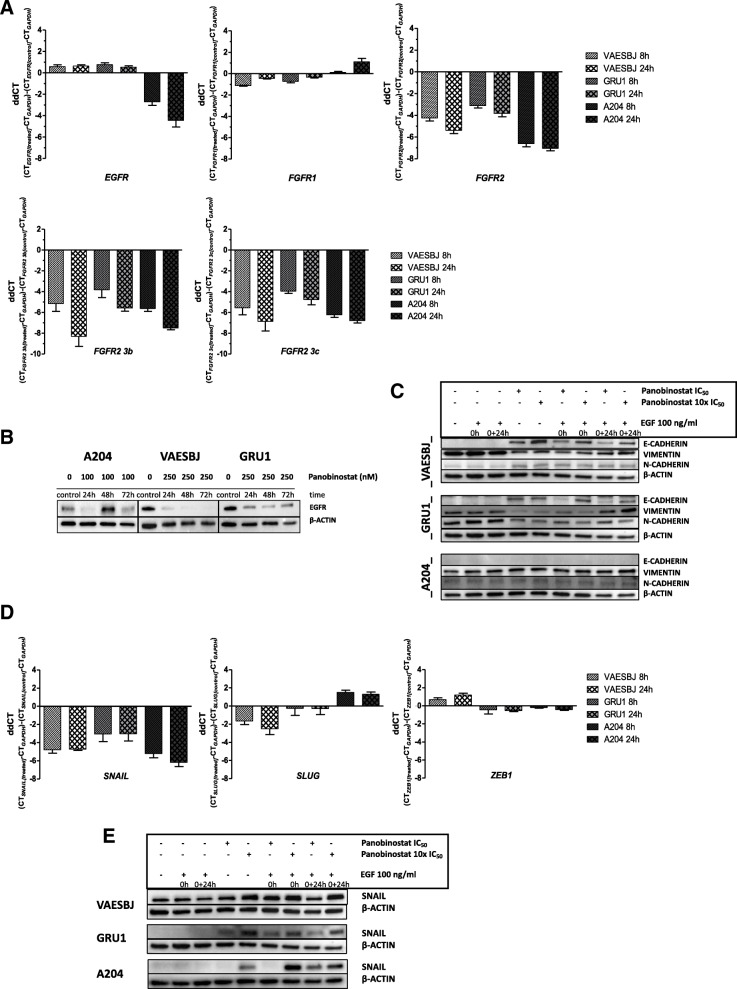
Panobinostat impacts on cellular differentiation via EGFR. **A** After treatment of cells with panobinostat (10xIC_50_: 100 nM for A204, and 250 nM for VAESBJ, GRU1) for 8 and 24 h, changes in mRNA expression levels for *EGFR*, *FGFR1* and *FGFR2* were determined in N ≥ 3 biological replicates in 3 technical replicates, normalized on *GAPDH* expression (top panel; graph represents means ± SEM; non-parametric Kruskal-Wallis test, *p < 0.0001* for *EGFR*, *FGFR1* and *p = 0.0001* for *FGFR2*). Similarly, expression changes of *FGFR2 3b* and *-3c* mRNA isoforms were determined (bottom panel; graph represents means ± SEM; non-parametric Kruskal-Wallis test, *p = 0.0024* (*FGFR2 3b*), *p = 0.0031* (*FGFR2 3c*)). **B** Protein expression of EGFR after panobinostat treatment for 24, 48 and 72 h. **C** Cells were maintained in MEM and treated with panobinostat at 10/100 nM for A204, and 25/250 nM for VAESBJ and GRU1 (IC_50_/10xIC_50_) for 24 h, and stimulated with EGF to determine expression of EMT-related proteins by Western blot. **D** Transcript expression changes of EMT transcription factors *SNAIL*, *SLUG* and *ZEB1* were measured after treatment of cells with panobinostat (10xIC_50_: 100 nM for A204, and 250 nM for VAESBJ, GRU1) for 8 and 24 h in N = 3 biological replicates in 3 technical replicates, normalized on *GAPDH* expression (graph represents means ± SEM; non-parametric Kruskal-Wallis test, *p = 0.0029* (*SNAIL*), *p = 0.01* (*SLUG*) and *p = 0.0048* (*ZEB1*)). **E** Serum-starved cells were treated with panobinostat at above indicated respective IC_50_ and 10xIC_50_ doses for 24 h, in the presence or without EGF, to confirm SNAIL upregulation on the protein level by Western blot

To assess if panobinostat modifies growth factor receptor expression, transcriptional changes for *FGFR1*, *FGFR2* and *EGFR* were determined after treatment. Panobinostat significantly changed expression levels of all three genes (Fig. 4A, top panel). In EGFR-overexpressing epithelioid sarcoma, *EGFR* transcript downregulation in VAESBJ and GRU1 corresponded to 66 and 64%, and 59 and 69% after 8 and 24 h, respectively. In contrast, A204 cells which only weakly express *EGFR*, strongly upregulated *EGFR* (6.5-fold after 8 h; 21.6-fold after 24 h). mRNA levels for *FGFR1* were slightly upregulated in epithelioid sarcoma (between 1.3 and 2.2-fold), and downregulated to 46% of the initial expression in A204. Interestingly, *FGFR2* mRNA expression was strongly augmented in all three cell lines, (19- and 42-fold (VAESBJ), 8.6- and 14.2-fold (GRU1) and 96.8- and 132.6-fold (A204) after 8 h and 24 h, respectively). Two *FGFR2* isoforms, *FGFR2 3b* and *FGFR2 3c*, have been shown to play a fundamental role in the cell’s orientation versus ‘epithelial’ or ‘mesenchymal’ differentiation, respectively. mRNA levels of both *FGFR2* isoforms were strongly upregulated following panobinostat (Fig. 4A, bottom panel). However, neither proliferation, nor migration/invasion, nor protein expression assays using corresponding ligands FGF2 and FGF7 for FGFR2 3b and 3c, respectively, led to conclusive results mounting evidence on their functional role (data not shown).

Following panobinostat treatment, EGFR protein was strongly reduced in GRU1 and almost undetectable in VAESBJ even after 72 h, whereas in A204 upregulation could be confirmed (Fig. 4B). Protein expression of key EMT proteins was determined to further evaluate the impact of panobinostat on cellular differentiation related to cancer invasiveness and metastases (Fig. 4C). In the two epithelioid sarcoma cell lines, E-CADHERIN was strongly induced by panobinostat in a dose-dependent manner and conversely, VIMENTIN expression was reduced. EGF clearly opposed this effect, relating EGFR signaling to epithelioid sarcoma dedifferentiation. N-CADHERIN was not inversely expressed to E-CADHERIN and independent of EGFR activation, keeping the mesenchymal-to-epithelial switch in an intermediate state. Scratch wound assays were performed to determine the effects of panobinostat on migration and invasion. Significant inhibition of migration and invasion was visible in GRU1 cells, but addition of EGF did not increase cellular mobility, in contrast to 10% FBS. (Additional file 1). To gain further insight into the process of EMT, expression changes of driving transcription factors were assessed by RT-qPCR after panobinostat treatment. Strongest upregulation occurred in *SNAIL*, the best characterized member, with 39.9- and 72.2-fold (A204), 27.8- and 26.4-fold (VAESBJ), and 8.3- and 8.1-fold (GRU1) after 8 and 24 h, respectively (Fig. 4D). SNAIL protein induction was dose-dependent in all three cell lines favoring the higher dose of panobinostat, suggesting a resistance strategy to panobinostat (Fig. 4E). Furthermore, EGF enhanced SNAIL expression in A204 cells in the presence of panobinostat, showing that panobinostat-induced *EGFR* upregulation is functionally employed in sustaining the mesenchymal phenotype. Collectively, these results show that panobinostat differentially modulates expression of growth factor receptors and induces epithelial differentiation in epithelioid sarcoma; but also, that panobinostat treatment provokes cellular salvage mechanisms to escape its antitumor effect.

### Panobinostat increases sensitivity to EGFR inhibition in both epithelioid sarcoma and rhabdoid tumor

**Fig. 5:**
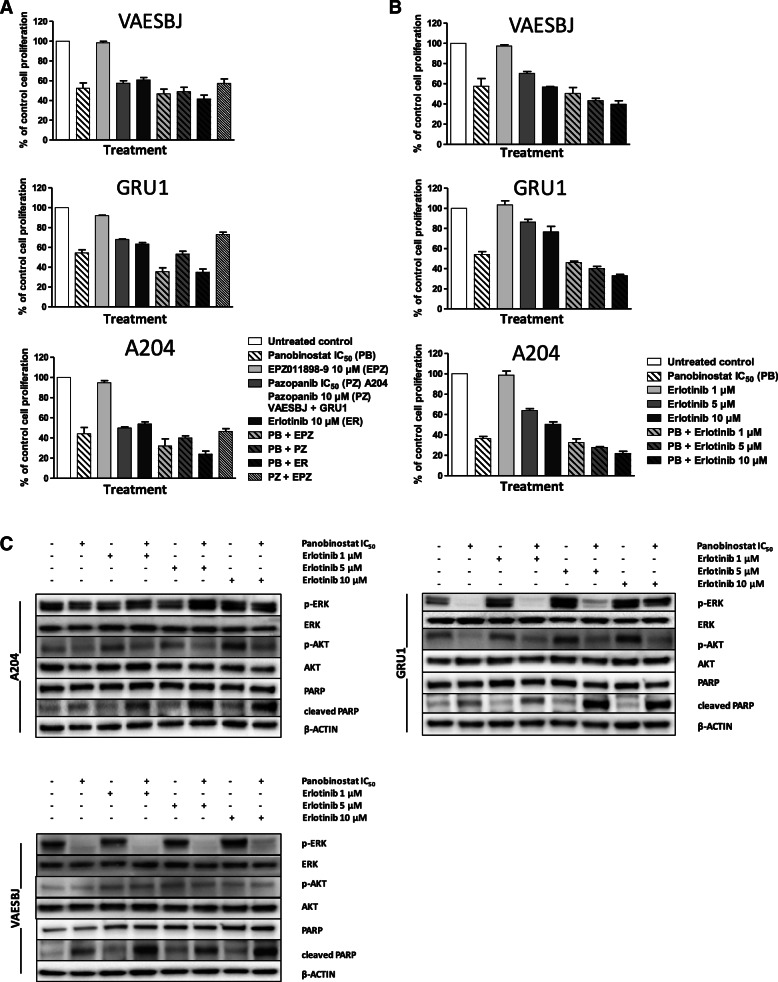
Combining panobinostat with inhibition of EGFR leads to enhanced apoptosis induction. **A** Effect of panobinostat at 16 nM for A204, 25 nM for VAESBJ and 60 nM for GRU1, corresponding to the IC_50_ as measured previously by MTS, with other anticancer agents on cell proliferation (427 nM of pazopanib for A204, as measured previously by MTS). Results determined by MTS assay in N ≥ 3 biological replicates (non-parametric Kruskal-Wallis test, *A204 p = 0.0037*, *VAESBJ p = 0.0013*, *GRU1 p = 0.0014*), graph represents means ± SEM. **B** Decrease of cell proliferation induced by panobinostat at the respective IC_50_ as indicated above with erlotinib at different dose levels after 72 h, by MTS in N ≥ 3 biological replicates (non-parametric Kruskal-Wallis test, *A204 p = 0.0027*, *VAESBJ p = 0.0051*, *GRU1 p < 0.0001*), graph represents means ± SEM. **C** Cells were treated for 24 h with panobinostat at the respective IC_50_ as indicated above in combination with different dose levels of erlotinib; activation of ERK and AKT as well as PARP cleavage was assessed by Western blot

To explore if the panobinostat-induced expression changes in growth factor receptors can be translated into active drug combinations, we screened panobinostat at respective IC_50_ doses with pazopanib, erlotinib and EPZ-011989-8 in cell proliferation tests, using IC_50_ doses or 10 μM if cells had been resistant. Details about dosing together with the compounds’ individual molecular targets are provided in additional file 7. For all three cell lines, panobinostat was confirmed being the most active compound. Enhanced combination effects were observed with erlotinib or EPZ011989–8 (Fig. 5A). Given our observations in the regulation of EGFR by panobinostat we explored further the combination of panobinostat to erlotinib at de-escalating doses of the latter, showing that even the lowest dose of 1 μM was slightly superior than panobinostat alone in all three cell lines; however, the most pronounced effect could be observed at the highest erlotinib dose of 10 μM (Fig. 5B). Consistently, combined treatment of panobinostat and erlotinib at the same dose levels increased apoptosis induction compared to panobinostat alone, as shown by Western blot after 24 h (Fig. 5C).

## Discussion

The fundamental contribution of epigenetic dysregulation to cancer development could be undoubtedly established as a surprisingly high number of human malignancies harbor mutations in genes involved in all layers steering epigenetic integrity. This holds true in particular for pediatric tumors which, due to different etiology and tissue origin, usually are characterized by a remarkably low number of somatic mutations, the best example of which includes rhabdoid tumor displaying solely *SMARCB1* recurrent mutations [28, 29]. In the present study, we showed the activity of several epigenetic drugs and receptor tyrosine kinase inhibitors in epithelioid sarcoma and rhabdoid tumor cell lines. Interestingly, we found only limited sensitivity to EZH2 inhibition in A204 rhabdoid tumor, and resistance in epithelioid sarcoma cells. In contrast, panobinostat uniformly showed the highest anti-proliferative effect in vitro. This observation was confirmed on a primary metastatic tumor cell line derived from a patient with progressive epithelioid sarcoma. Further, treatment of the highly rapid growing VAESBJ xenograft model resulted in significant tumor growth inhibition. Overexpression of several HDACs has been shown in ATRT and rhabdoid tumor cell lines whereas data are lacking for epithelioid sarcoma [30]. Panobinostat is a potent pan-HDAC inhibitor inhibiting all eleven HDACs with IC_50_ values mostly in the low nanomolar range, and it is about 10-fold more potent than vorinostat [31]. Although considerable sensitivity was observed for vorinostat and mocetinostat as well, the high sensitivity towards panobinostat might be related to broad HDAC inhibition replacing more sufficiently the global defect chromatin organization caused by INI1 alteration. Conditional biallelic inactivation of *Smarcb1* in vivo causes rapid onset of aggressive tumors analogous to human rhabdoid tumor and T-cell lymphomas, much faster than in other tumor suppressor inactivation mouse models, including *Tp53* [32]. Interestingly, GRU1 cells which retain INI1 expression showed the most resistant profile regarding all epigenetic agents tested in our study.

Functionally, next to the strong cytotoxic effect through induction of apoptotic cell death, HDAC inhibition led to significant *EGFR* downregulation in epithelioid sarcoma which is one of the best characterized oncogenes, and its activation is tightly related to poor survival and cancer progression [33]. To our knowledge, HDAC inhibitor-mediated downregulation has been described only for classical epithelial tumors so far, such as colon and lung cancer [34, 35]. The type of genetic alterations leading to constitutive activation of *EGFR*, however, tend to show disease-specific predilection and are closely connected to predicting drug responses. Similar to epithelioid sarcoma, EGFR overexpression is present in certain epithelial cancers but overexpression alone does not predict treatment response to EGFR inhibition, in contrast to kinase domain mutations, e.g. in exons 18–21 of *EGFR* in NSCLC patients [23, 36, 37]. This is supported by our results showing erlotinib not reducing proliferation in epithelioid sarcoma cells, and consistent with the absence of *EGFR* tyrosine kinase mutations in this malignancy [23]. Rather, EGFR contributes to mesenchymal dedifferentiation as panobinostat induced expression of key EMT protein E-CADHERIN, and inversely led to VIMENTIN repression, which could be challenged by EGFR activation. In concordance with this, mobility was inhibited in GRU1 cells. On the transcriptional level, *EGFR* expression was reduced by around one third, whereas on the protein level EGFR expression was markedly reduced in GRU1 cells, and virtually absent in VAESBJ cells. This discrepancy might be attributable to the fact that HDAC inhibition increases acetylation not only on histones but also on non-histone proteins, one of which is HSP90 leading to inactivation of its chaperoning function of several proteins, including EGFR [38].

In rhabdoid tumors, previous reports have associated both cranial and extracranial tumors to elevated EGFR expression, activation and successful pharmacological inhibition, and more recently, in a INI1-dependent context [19, 39]. In A204 cells, we detected a rather low mRNA and protein expression of EGFR which was strongly upregulated by panobinostat. This particular cell line has been related to INI1-dependent FGFR1 overexpression and successful inhibition by NVP-BGJ398, a specific FGFR inhibitor [22]. Newer studies have also highlighted the necessity to perform dual growth factor receptor inhibition, including PDGFRA and FGFR1 or FGFR2, depending on the cell lines used [20, 21]. The observed in vitro sensitivity of A204 towards pazopanib or regorafenib, which comprise these targets, confirms previous reports and was further supported by significant antitumor activity and objective tumor responses of regorafenib in vivo. Nevertheless, as only one rhabdoid tumor cell line was used in this study, primarily to compare the results of epithelioid sarcoma to a different tumor with a similar genetic background, these findings should be confirmed in further models. In contrast in FGFR1 overexpressing epithelioid sarcoma, regorafenib, as well as pazopanib, the latter being approved for the treatment of advanced soft tissue sarcomas since 2012, exhibited limited or no sensitivity, and activated the MAPK and PI3K/AKT/mTOR signaling pathways in both cell lines in vitro. Regorafenib treatment induced significant tumor growth inhibition of VAESBJ xenografts which could likely be assigned to the antiangiogenic effect of regorafenib, as described by our group previously [22, 26]. These observations are aligned with the report from a recent retrospective trial evaluating pazopanib in 18 patients with epithelioid sarcoma which reported no objective responses but an inferior outcome as compared to patients that had received chemotherapy [40]. Remarkably however, our own clinical experience includes a young adult patient with metastatic disease who the primary cells used in this study were derived from. This patient underwent complete remission by pazopanib, ongoing for 3 years [27]. She had received multiple preceding treatments including surgery, cryotherapy, conventional chemotherapy, erlotinib in combination with rapamycin, and lastly EZH2 inhibitor tazemetostat (NCT02601937). Possibly, these treatments had a modifying effect on the tumor in this individual case which is related to the treatment response to pazopanib. Our results underline the necessity to explore drug responses in different contexts, as different models are characterized by strengths and weaknesses.

We further observed strong induction of *FGFR2* in all three cell lines and, surprisingly, of both its isoforms 3b and 3c which are important players in EMT, executing opposing functions [41]. Functional exploration however did not conclusively support FGFR2 3b or FGFR2 3c triggering either epithelial or mesenchymal differentiation, respectively, leaving the role of *FGFR2* upregulation elusive.

Unexpectedly, drug combination tests revealed that panobinostat rendered all three cell lines cells more susceptible towards erlotinib. For epithelioid sarcoma cell lines, this might be related to E-CADHERIN re-expression which has been correlated to restore EGFR-TKI sensitivity in epithelial cancers, including both resistant *EGFR*-mutated and -non-mutated lung cancer [42, 43]. As, unsurprisingly, we did not detect an inducible mesenchymal-to-epithelial shift in A204 cells due to the non-epithelial origin of rhabdoid tumor, the strong induction of *EGFR* by HDAC inhibition rather represents a resistance mechanism, leading to a ‘kinase switch’ to sustain mesenchymal dedifferentiation. Further, in all three cell lines tested we observed a dose-dependent upregulation of *SNAIL*, the best characterized transcription factor driving the mesenchymal phenotype by activating EMT-responsive genes. Although HDAC inhibitors selectively target cancer cells and change gene expression only in 2–8%, they might nevertheless exert desired as well as undesired effects [44]. Increased deregulation of already activated oncogenic signaling pathways after single HDAC inhibitor treatment has been observed, requiring specific drug combinations to target these pathways [14, 30]. Upregulation of EMT transcription factors following HDAC inhibition has been described in the context of carcinoma cell lines [45, 46]. In VAESBJ and A204 cells, upregulation on both transcription and protein level was stronger than in GRU1 cells, in line with panobinostat inhibiting migration/invasion most potently in GRU1 cells. But in contrast to epithelioid sarcoma, induction of SNAIL by panobinostat in A204 cells was further enhanced when stimulated with EGF. Interestingly, in cisplatin-resistant ATRT, SNAIL expression has been shown to be directly regulated by STAT3 which, in turn, is a downstream target of various cytokines and tyrosine kinases, including EGFR [47]. With our results we provide evidence that EGFR serves rather as a differentiation- than a tumor growth factor and, that the discordant EGFR regulation in epithelioid versus rhabdoid tumor cell lines by panobinostat is mechanistically exploited differently. In EGFR-overexpressing epithelioid sarcoma, the combination of erlotinib and panobinostat might have further hampered residual oncogenic EGFR-mediated signaling and thereby reduced cell proliferation and -survival, in addition to largely reduced EGFR levels by panobinostat alone. In contrast in the rhabdoid tumor, results point at employment of increased EGFR expression as resistance mechanism. EGFR has been shown to be expressed at higher levels in INI1-deficient than -competent tumors [19]; we have shown that EGFR expression can be augmented via panobinostat treatment, and that factors which conserve the mesenchymal phenotype can be promoted by stimulating EGFR by its respective ligand. Therefore, it is conceivable that eliciting EGFR-mediated reponses also renders cells more susceptible to inhibition by targeting its kinase domain. Furthermore, as panobinostat has been shown to destabilize EGFR, for both tumor types it cannot be excluded that this also leads to conformational changes in the kinase domain which increase sensitivity to erlotinib.

However, our observations are limited to cell lines, and it would be crucial to extend and confirm our results in both more well characterized cell lines as well as in representative patient-derived xenografts. For rhabdoid tumors in particular, models should be chosen which comprise both also ATRT and rhabdoid tumors of the kidney, and also taking into account the molecular dissection of ATRT into three distinct epigenetic subgroups [48].

From a clinical point of view, there is a precarious gap between promising preclinical data and disappointing lack of single agent efficacy in patients with solid cancers, an example of which is diffuse intrinsic pontine glioma (DIPG), a pediatric tumor with dismal outcome. Panobinostat alone (NCT02717455) or in combination with a proteasome inhibitor (NCT04341311) has entered clinical investigation for this entity based on promising preclinical data [49]. However, later studies yielded discordant results in vivo despite the fact that in a genetically-engineered mouse model supposedly active concentrations could be achieved in the brainstem tumor, as opposed to the normal cortex [49, 50]. The relatively intact blood-brain barrier (BBB) that characterizes DIPG might be a main factor that up to present, preclinically effective treatments could not be translated into clinics. To bypass the BBB, currently convection-enhanced delivery (CED) for panobinostat has been brought forward [49, 51]. The resulting agent MTX110 is currently under clinical investigation for both DIPG (NCT03566199) and medulloblastoma (NCT04315065). Various HDAC inhibitors like entinostat alone (NCT02780804) and in combination (INFORM 2; NCT03838042) are now being explored in pediatric cancers and data are awaited for these. Furthermore, regorafenib as various other multi-tyrosine kinase inhibitors are currently introduced into sarcoma treatment combined with chemotherapy whereas selective FGFR inhibitor have only started recently in phase 1 trials (i.e. erdafitinib NCT04083976 or futibatinib in our AcSe-ESMART NCT02813135).

This study was dedicated to decipher cellular and regulatory effects of HDAC inhibitors in two aggressive sarcoma types. Next to the great cytotoxic potential of panobinostat at low nanomolar concentrations in these tumors, we show to the best of our knowledge for the first time that HDAC-mediated growth factor receptor regulation, EGFR in particular, is not an exclusive feature to epithelial-derived tumors. We delineate that EGFR has important implications in cellular dedifferentiation rather than proliferation in both genetically linked tumor types. Intrinsic EGFR overexpression in epithelioid sarcoma maintains the cell’s mesenchymal traits which are in part reversible by panobinostat. In rhabdoid tumor cell line A204, the induced EGFR upregulation serves to resist the differentiating potential of HDAC inhibitors. Therapeutic utility of HDAC inhibitors in solid tumors might be by far better exploitable by combining HDAC inhibitors with other agents which circumvent cellular salvage mechanisms. Their relevance, however, should be validated in further suitable models including patient-derived models. Better mechanistic understanding in particular tissue contexts is indispensable and will render drug combinations with HDAC inhibitors increasingly attractive as reflected by the emerging number of ongoing clinical trials, with other epigenetic drugs, with targeted therapy, with cytotoxic chemotherapy/radiotherapy, and lastly, with immunotherapy based on the reprogramming and drug-sensitizing potential of epigenetic modulation [52, 53].

## Conclusion

In this study, INI1-altered sarcoma types were highly sensitive to pan-HDAC inhibitor panobinostat in vitro. In addition to its antiproliferative and cell death-promoting effect, panobinostat differentially regulated expression of *EGFR* in epithelioid sarcoma and rhabdoid tumor which, according to our knowledge, has been only described in carcinomas so far. The role of EGFR in these tumors implicates cellular differentiation rather than proliferation. Exploited therapeutically, the combination of panobinostat with EGFR inhibitor erlotinib was shown to be superior. Further validating studies in a larger panel of cell lines and patient-derived tumor models should be performed and will help to expand the limited efficacy of HDAC inhibitors as single agents in solid tumors, to provide new therapeutic approaches in these devastating malignancies.

## Supplementary Information

**Additional file 1. **Results of migration and invasion assays after treatment with panobinostat. Serum-starved cells were treated with panobinostat at indicated concentrations plus EGF 100 ng/ml or FBS 10% to perform migration/invasion assays (means ± SEM, *N* ≥ 3 replicates, non-parametric Kruskal-Wallis test). Significant migration inhibition in GRU1, *p = 0.0018, p = 0.0027 and p = 0.0132* in serum-starved, EGF- and FBS 10%-stimulated condition, respectively; significant invasion inhibition in GRU1, *p = 0.0069*, in EGF-stimulated condition.

**Additional file 2.** The uncropped Western Blots shown as part of Fig. 1.

**Additional file 3.** The uncropped Western Blots shown as part of Fig. 2.

**Additional file 4.** The uncropped Western Blots shown as part of Fig. 3.

**Additional file 5.** The uncropped Western Blots shown as part of Fig. 4.

**Additional file 6.** The uncropped Western Blots shown as part of Fig. 5.

**Additional file 7.** Details about treatment combinations shown in Fig. 5A.

## Data Availability

The datasets generated and analyzed in the current study are available from the corresponding author on reasonable request.

## Abbreviations

APAFIS: Autorisation de projet utilisant des animaux à des fins
ATCC: American Type Culture Collection
ATP: Adenosine triphosphate
ATRT: Atypical teratoid rhabdoid tumor
CO: Control
CT: Cycle threshold
d: Delta
DMEM: Dulbecco‘s Modified Eagle Medium
DMSO: dimethylsulfoxide
DNA: Desoxyribonucleic acid
DSMZ: Deutsche Sammlung von Mikroorgansimen und Zellkulturen
EA: Early apoptosis
EGF: Epidermal Growth Factor
EGFR: Epidermal Growth Factor Receptor
EMT: Epithelial-to-Mesenchymal transition
EZH2: Enhancer Of Zeste 2 Polycomb Repressive Complex 2 Subunit
FACS: Fluorescence-activated cell-sorting
FBS: Fetal bovine serum
FGF: Fibroblast Growth Factor
FGFR1: Fibroblast Growth Factor Receptor 1
FGFR2: Fibroblast Growth Factor Receptor 2
GAPDH: Glyceraldehyde-3-Phosphate Dehydrogenase
HDAC: Histone deacetylase
IC: Inhibitory concentration
INI1: Integrase Interactor 1 Protein
IQR: Interquartile range
KGF: Keratinocyte Growth Factor
LA: Late apoptosis
MEM: Minimal Essential Medium
MENESR: Ministère de l’éducation nationale, de l’enseignement supérieur et de la recherche
mRNA: Messenger ribonucleic acid
mTOR: Mechanistic target Of Rapamycin Kinase
MTS: 3-(4,5-dimethylthiazol-2-yl)-5-(3-carboxymethoxyphenyl)-2-(4-sulfophenyl)-2H-tetrazolium)
N: Necrosis
NCT: National Clinical Trial
PBS: Phosphate Buffered Saline
PDGFRA: Platelet-Derived Growth Factor Receptor A
PRC2: Polycomb repressive complex 2
RNA: Ribonucleic acid
RT-qPCR: Reverse transcription quantitative real-time polymerase chain reaction
SEM: Standard error of mean
SMARCB1: SWI/SNF Related, Matrix Associated, Actin Dependent Regulator Of Chromatin, Subfamily B, Member 1
SWI/SNF: SWItch/Sucrose Non-Fermentable
TGD: Tumor growth delay
TGI: Tumor growth inhibition
TKI: Multi-tyrosine kinase inhibitors
TP53: Tumor Protein P53
ZEB1: Zinc Finger E-Box Binding Homeobox 1
