# Supplementary material for: Histone deacetylase inhibitor panobinostat induces antitumor activity in epithelioid sarcoma and rhabdoid tumor by growth factor receptor modulation
Source: BMC Cancer. 2021 Jul 20;21:833. doi: 10.1186/s12885-021-08579-w (PMC8290558; doi:10.1186/s12885-021-08579-w)

# Histone deacetylase inhibitor panobinostat induces antitumor activity in epithelioid sarcoma and rhabdoid tumor by growth factor receptor modulation

Anne Catherine Harttrampf, Maria Eugenia Marques da Costa, Aline Renoult, Estelle Daudigeos-Dubus, Birgit Geoerger

**Additional file 1:** Results of migration and invasion assays after treatment with panobinostat.

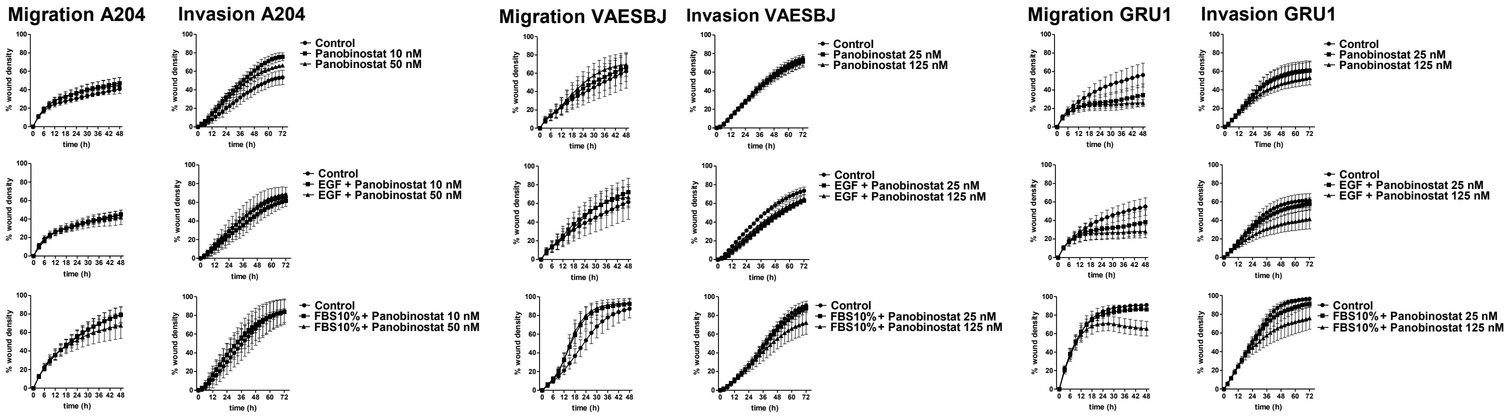

Supplement: Supplementary file 1 — Additional file 1. Results of migration and invasion assays after treatment with panobinostat. Serum-starved cells were treated with panobinostat at indicated concentrations plus EGF 100 ng/ml or FBS 10% to perform migration/invasion assays (means ± SEM, N ≥ 3 replicates, non-parametric Kruskal-Wallis test). Significant migration inhibition in GRU1, p = 0.0018, p = 0.0027 and p = 0.0132 in serum-starved, EGF- and FBS 10%-stimulated condition, respectively; significant invasion inhibition in GRU1, p = 0.0069, in EGF-stimulated condition. [file 12885_2021_8579_MOESM1_ESM.pdf]
