# Supplementary material for: Histone deacetylase inhibitor panobinostat induces antitumor activity in epithelioid sarcoma and rhabdoid tumor by growth factor receptor modulation
Source: BMC Cancer. 2021 Jul 20;21:833. doi: 10.1186/s12885-021-08579-w (PMC8290558; doi:10.1186/s12885-021-08579-w)

Histone deacetylase inhibitor panobinostat induces antitumor activity in epithelioid sarcoma and rhabdoid tumor by growth factor receptor modulation

Anne Catherine Harttrampf, Maria Eugenia Marques da Costa, Aline Renoult, Estelle Daudigeos-Dubus, Birgit Geoerger

Additional file 2: The uncropped Western Blots shown as part of Figure 1.

Figure 1B: the 3 middle lanes from the 5 lanes shown refer to A204, VAESBJ, GRU1 (left to right)

Figure 1B: SMARCB1 (45 kD)

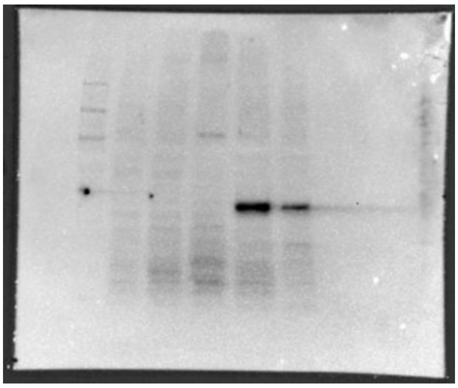

Figure 1B: β-Actin (45 kD)

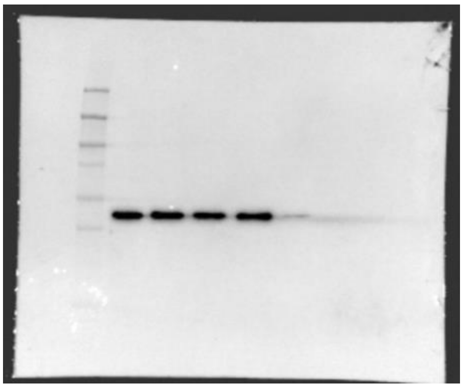

Figure 1C: Acetyl-Histone 4 (10 kD)

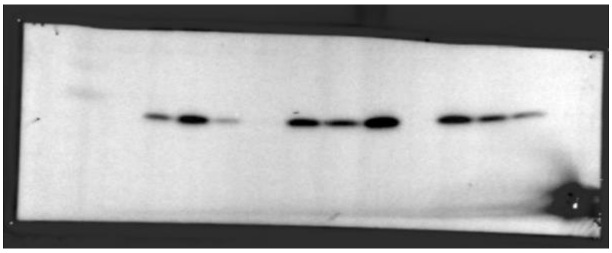

Figure 1C: p21 (21 kD)

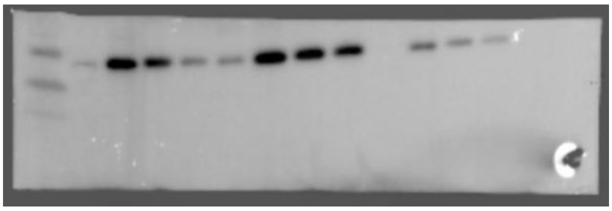

Figure 1C: β-Actin (45 kD)

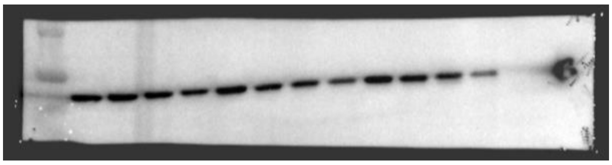

Figure 1D: p-ERK (42 & 44 kD)

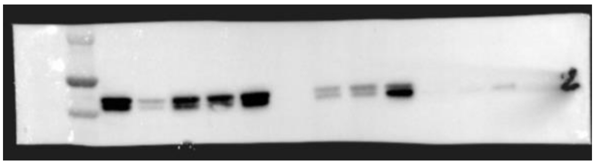

Figure 1D: ERK (42 & 44 kD)

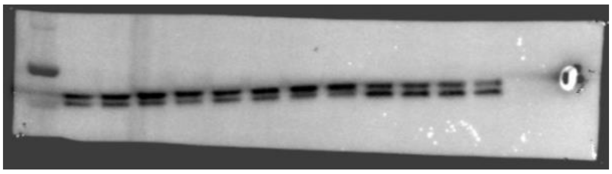

Figure 1D: p-AKT (60 kD, upper band)

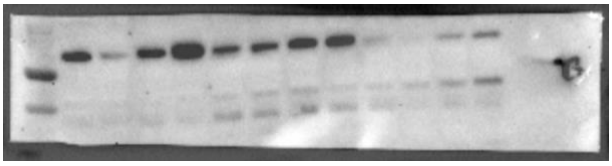

Figure 1D: AKT (60 kD, upper band)

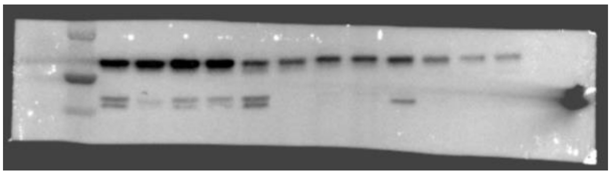

Figure 1D: β-Actin (45 kD)

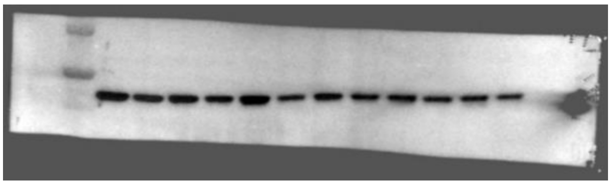

Figure 1F: PARP (110 kD, top bands) & cleaved PARP (89 kD, second bands from the top)

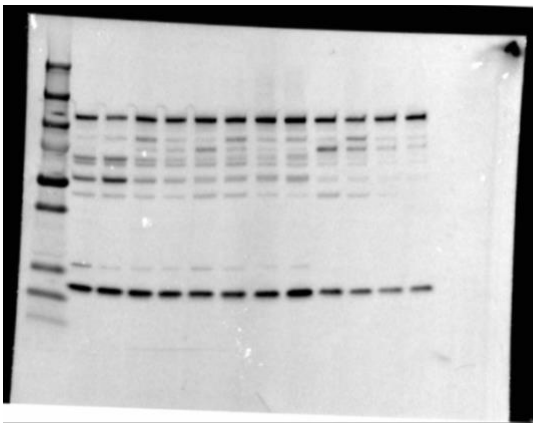

Figure 1F: Caspase 3 (35 kD)

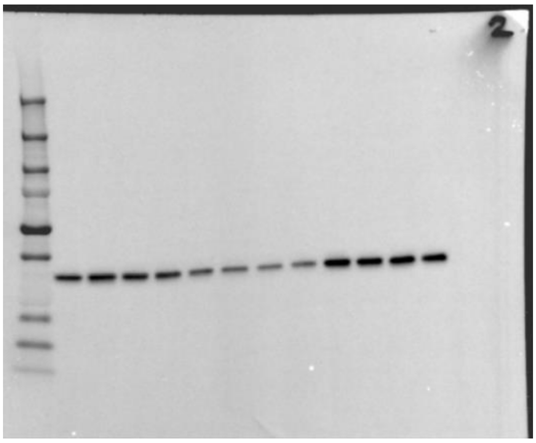

Figure 1F: cleaved Caspase 3 (17 kD, lower band)

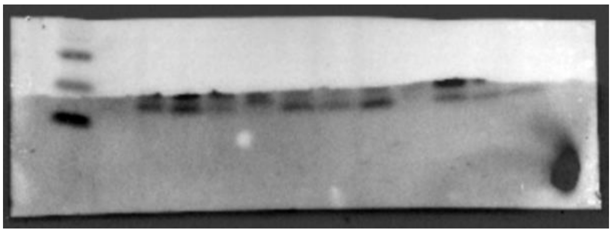

Figure 1F: β-Actin (45 kD)

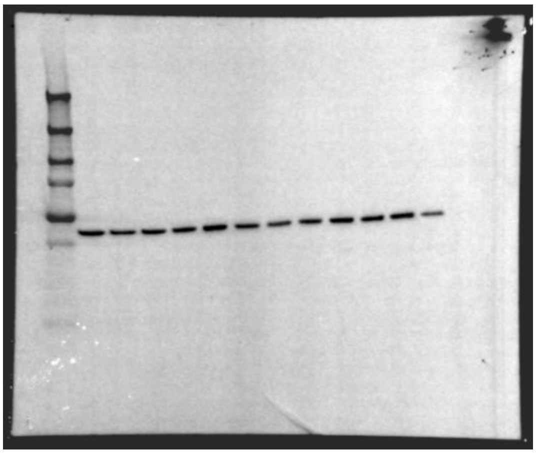

Supplement: Supplementary file 2 — Additional file 2. The uncropped Western Blots shown as part of Fig. 1. [file 12885_2021_8579_MOESM2_ESM.pdf]
