# Supplementary material for: Histone deacetylase inhibitor panobinostat induces antitumor activity in epithelioid sarcoma and rhabdoid tumor by growth factor receptor modulation
Source: BMC Cancer. 2021 Jul 20;21:833. doi: 10.1186/s12885-021-08579-w (PMC8290558; doi:10.1186/s12885-021-08579-w)

Histone deacetylase inhibitor panobinostat induces antitumor activity in epithelioid sarcoma and rhabdoid tumor by growth factor receptor modulation

Anne Catherine Harttrampf, Maria Eugenia Marques da Costa, Aline Renoult, Estelle Daudigeos-Dubus, Birgit Geoerger

Additional file 3: The uncropped Western Blots shown as part of Figure 2.

Figure 2D Regorafenib: order on gel A204 (4 bands left), VAESBJ (4 bands in the middle), GRU1 (4 bands right) if not indicated separately

Figure 2D: p-AKT (60 kD)

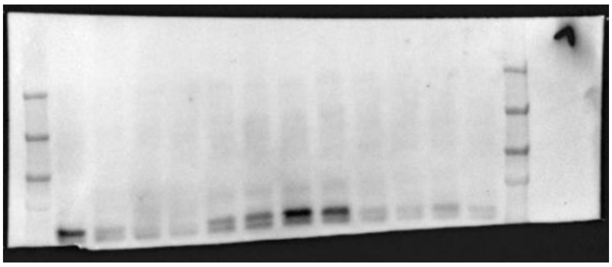

Figure 2D: AKT (60 kD)

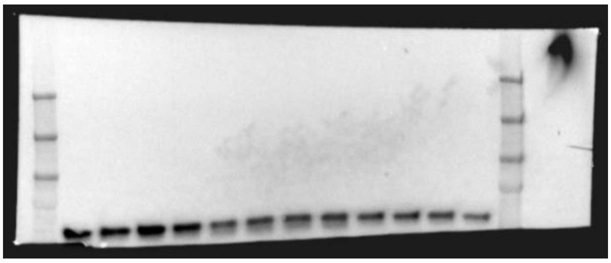

Figure 2D: p-ERK (42 & 44 kD, A204)

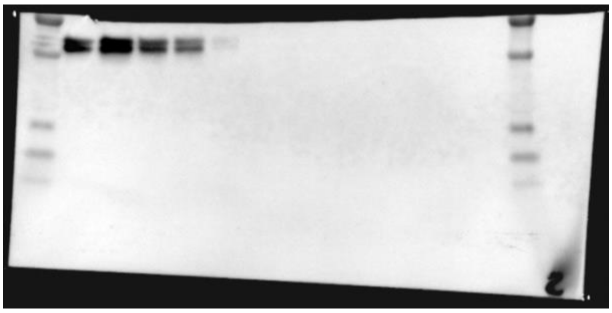

Figure 2D: p-ERK (42 & 44 kD, VAESBJ left -, GRU1 right 4 bands)

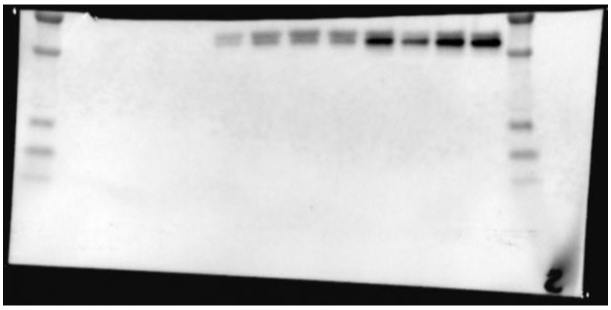

Figure 2D: ERK (42 & 44 kD)

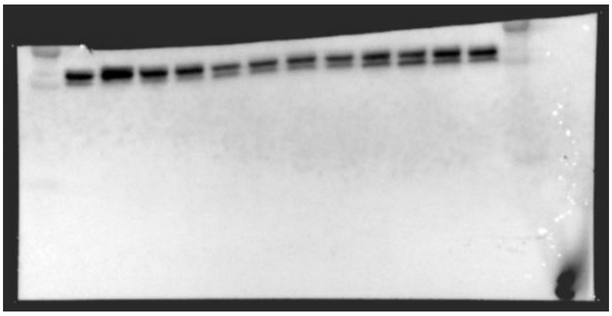

Figure 2D: β-Actin (45 kD)

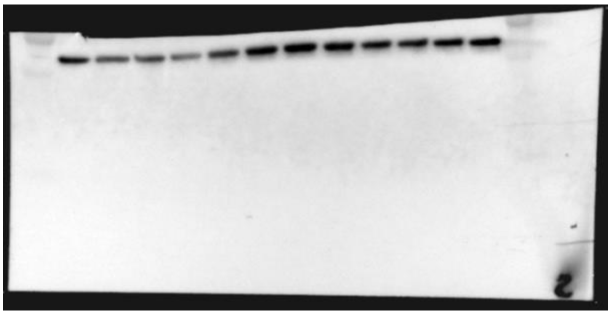

Figure 2D Pazopanib: order (4 bands each) > VAESBJ left/ GRU1 middle/ A204 right

Figure 2D: p-AKT (60 kD)

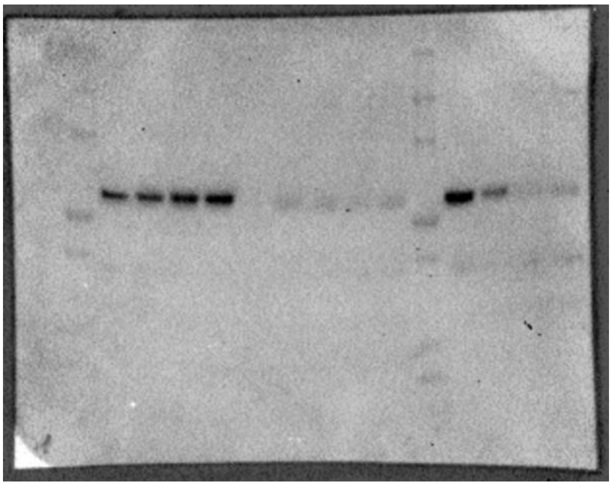

Figure 2D: AKT (60 kD, upper bands)

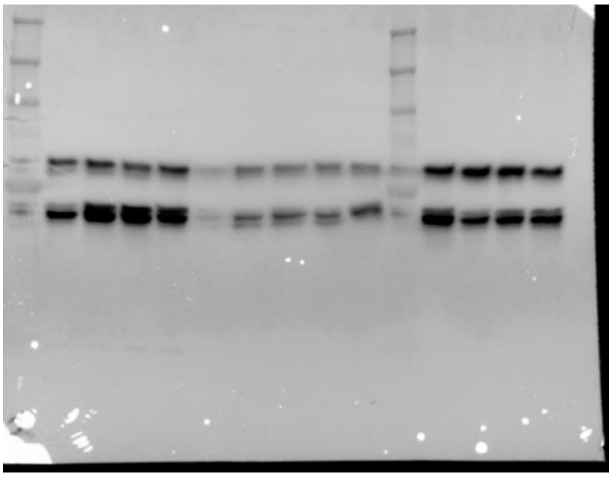

Figure 2D: p-ERK (42 & 44 kD)

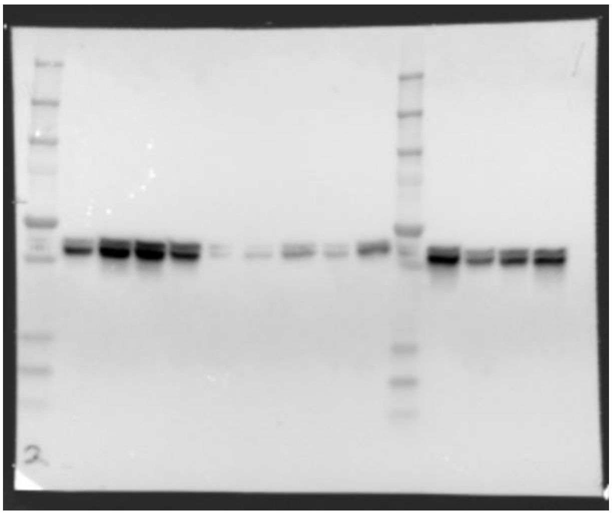

Figure 2D: ERK (42 & 44 kD, lower bands)

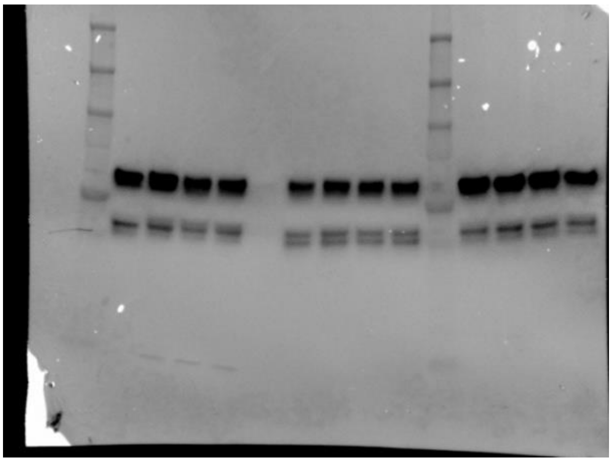

Figure 2D: β-Actin (45 kD, lower bands)

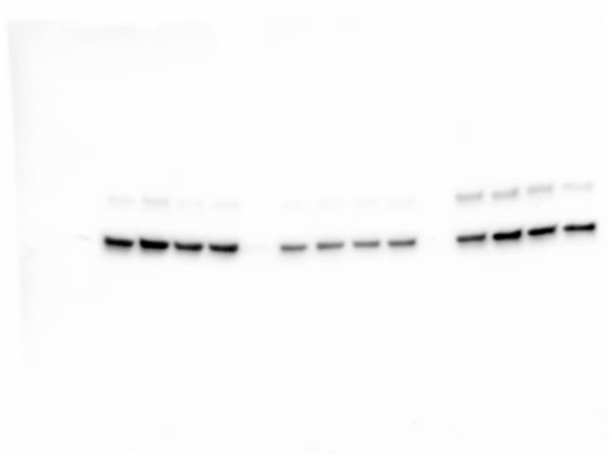

Supplement: Supplementary file 3 — Additional file 3. The uncropped Western Blots shown as part of Fig. 2. [file 12885_2021_8579_MOESM3_ESM.pdf]
