# Supplementary material for: Histone deacetylase inhibitor panobinostat induces antitumor activity in epithelioid sarcoma and rhabdoid tumor by growth factor receptor modulation
Source: BMC Cancer. 2021 Jul 20;21:833. doi: 10.1186/s12885-021-08579-w (PMC8290558; doi:10.1186/s12885-021-08579-w)

Histone deacetylase inhibitor panobinostat induces antitumor activity in epithelioid sarcoma and rhabdoid tumor by growth factor receptor modulation

Anne Catherine Harttrampf, Maria Eugenia Marques da Costa, Aline Renoult, Estelle Daudigeos-Dubus, Birgit Geoerger

Additional file 4: The uncropped Western Blots shown as part of Figure 3.

Figure 3C Panobinostat *i.p.*: order on gel > control (3 bands left), 12 mg/kg (4 bands in the middle), 8 mg/kg (4 bands right)

Figure 3C: p-AKT (60 kD, upper band)

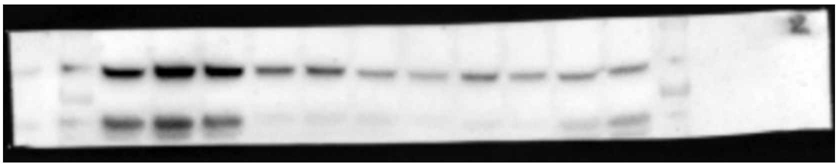

Figure 3C: AKT (60 kD, upper band)

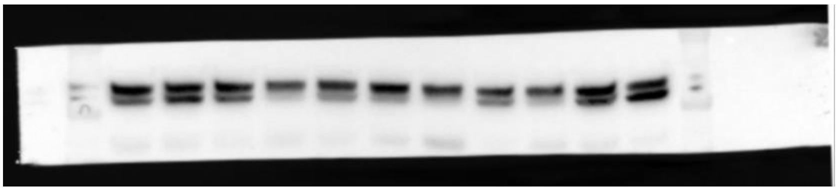

Figure 3C: p-ERK (42 & 44 kD)

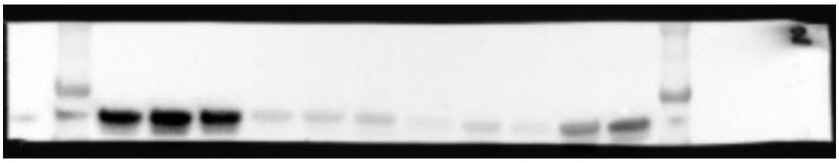

Figure 3C: ERK (42 & 44 kD, lower band)

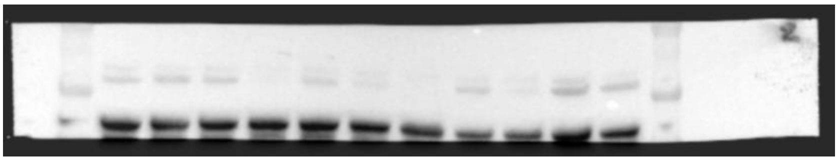

Figure 3C: p-SHC (50 & 55 kD)

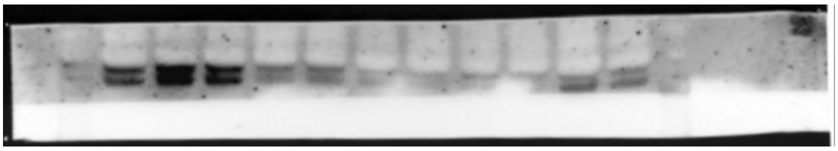

Figure 3C: β-Actin (45 kD)

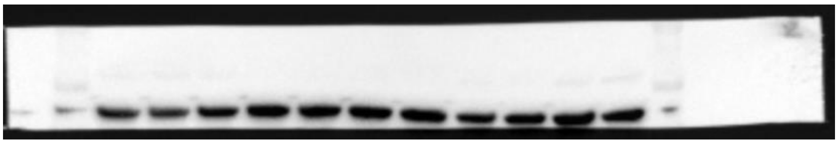

Supplement: Supplementary file 4 — Additional file 4. The uncropped Western Blots shown as part of Fig. 3. [file 12885_2021_8579_MOESM4_ESM.pdf]
