# Supplementary material for: Histone deacetylase inhibitor panobinostat induces antitumor activity in epithelioid sarcoma and rhabdoid tumor by growth factor receptor modulation
Source: BMC Cancer. 2021 Jul 20;21:833. doi: 10.1186/s12885-021-08579-w (PMC8290558; doi:10.1186/s12885-021-08579-w)

Histone deacetylase inhibitor panobinostat induces antitumor activity in epithelioid sarcoma and rhabdoid tumor by growth factor receptor modulation

Anne Catherine Harttrampf, Maria Eugenia Marques da Costa, Aline Renoult, Estelle Daudigeos-Dubus, Birgit Geoerger

Additional file 5: The uncropped Western Blots shown as part of Figure 4.

Figure 4B

Figure 4B: EGFR (175 kD, A204)

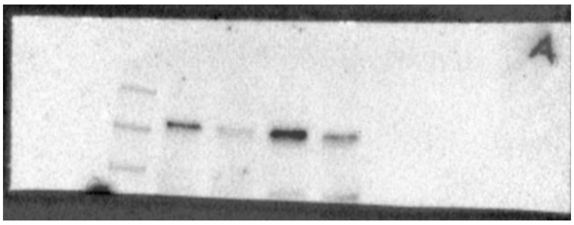

Figure 4B: (175 kD, VAESBJ left 4 bands, GRU1 right four bands)

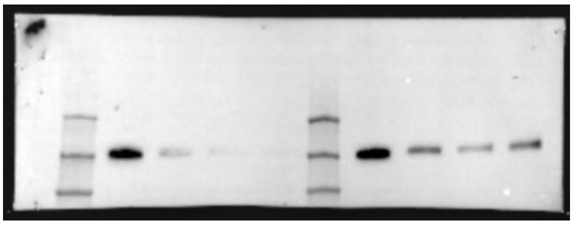

Figure 4B: β-Actin (45 kD, A204, top band)

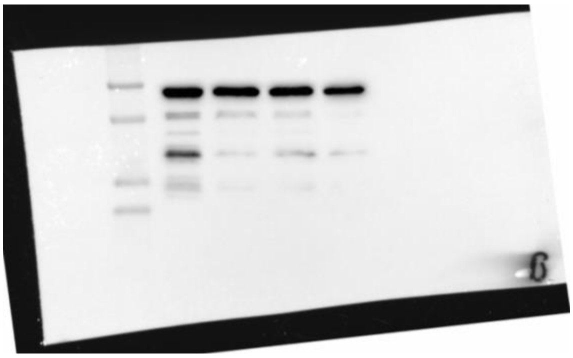

Figure 4B: β-Actin (45 kD, VAESBJ left 4 bands, GRU1 right four bands)

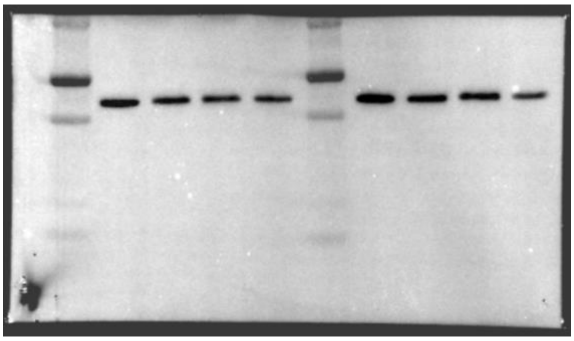

Figure 4C

VAESBJ

Figure 4C: E-Cadherin (135 kD, lower bands)

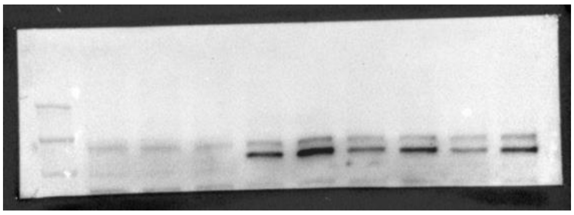

Figure 4C: Vimentin (53 kD, middle bands)

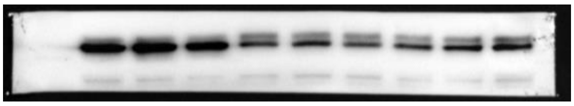

Figure 4C: N-Cadherin (140 kD, lower bands)

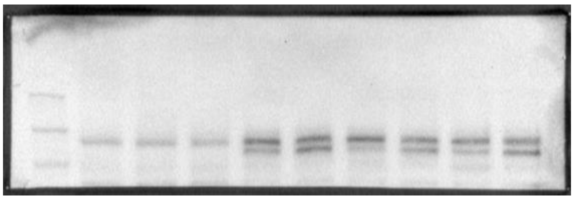

Figure 4C: β-Actin (45 kD)

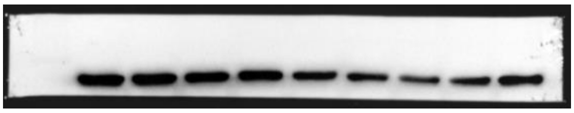

GRU1

Figure 4C: E-Cadherin (135 kD, 2nd row from top)

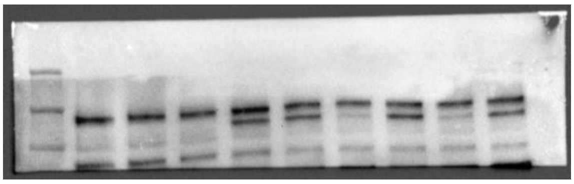

Figure 4C: Vimentin (53 kD)

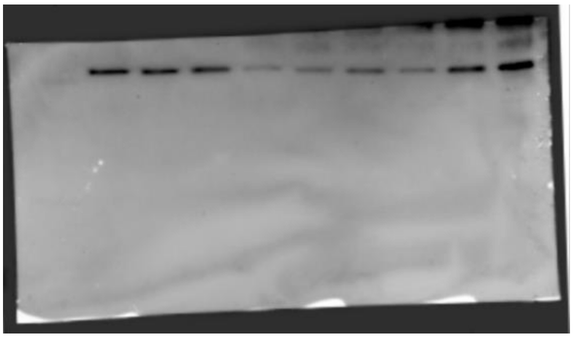

Figure 4C: N-Cadherin (140 kD)

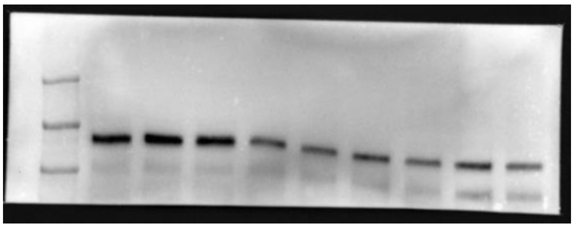

Figure 4C: β-Actin (45 kD)

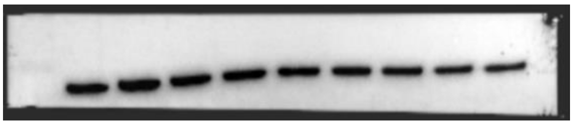

<u>A204</u>

Figure 4C: E-Cadherin (135 kD)

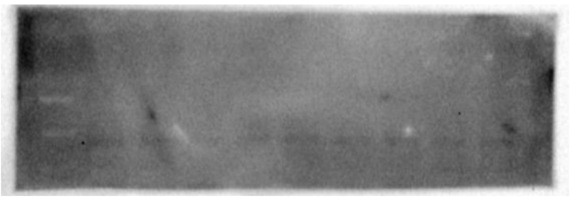

Figure 4C: Vimentin (53 kD)

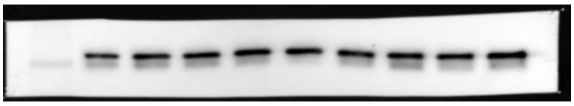

Figure 4C: N-Cadherin (140 kD, lowest bands)

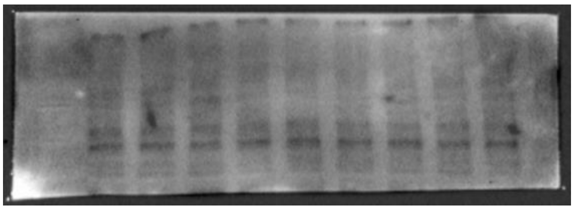

Figure 4C: β-Actin (45 kD)

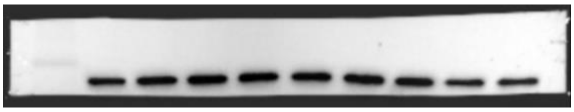

Figure 4E

VAESBJ

Figure 4E: Snail (29 kD)

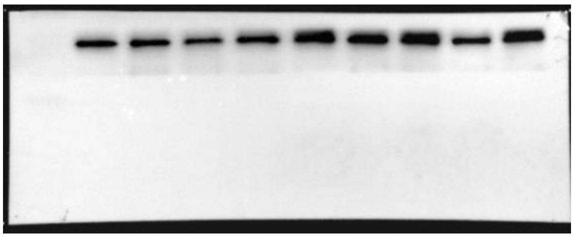

Figure 4E: β-Actin (45 kD)

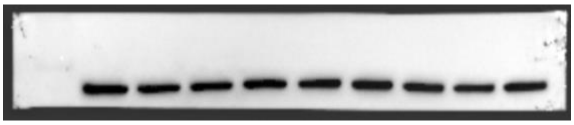

GRU1-1

Figure 4E: Snail (29 kD)

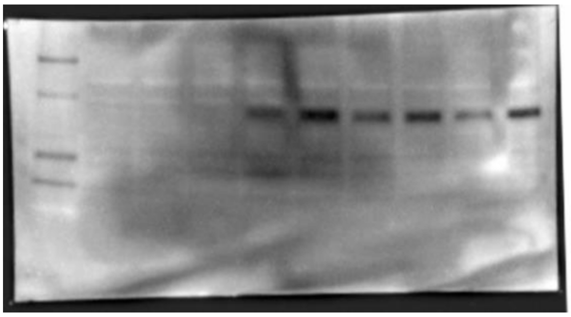

Figure 4E: β-Actin (45 kD)

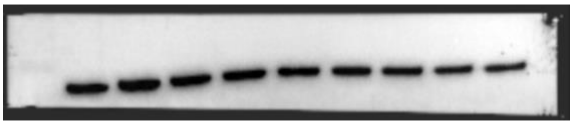

A204

Figure 4E: Snail (29 kD)

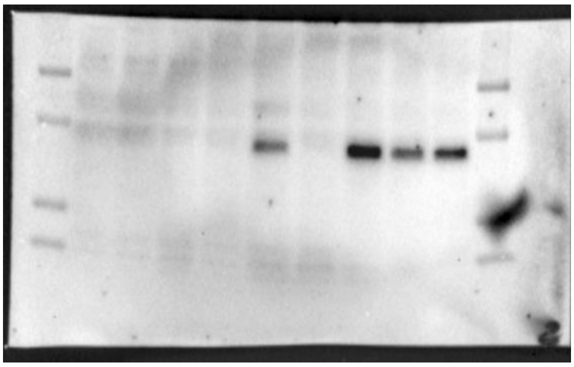

Figure 4E: β-Actin (45 kD, upper bands)

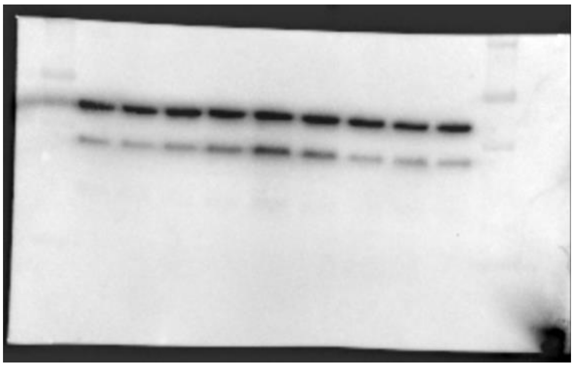

Supplement: Supplementary file 5 — Additional file 5. The uncropped Western Blots shown as part of Fig. 4. [file 12885_2021_8579_MOESM5_ESM.pdf]
