# Supplementary material for: Histone deacetylase inhibitor panobinostat induces antitumor activity in epithelioid sarcoma and rhabdoid tumor by growth factor receptor modulation
Source: BMC Cancer. 2021 Jul 20;21:833. doi: 10.1186/s12885-021-08579-w (PMC8290558; doi:10.1186/s12885-021-08579-w)

Histone deacetylase inhibitor panobinostat induces antitumor activity in epithelioid sarcoma and rhabdoid tumor by growth factor receptor modulation

Anne Catherine Harttrampf, Maria Eugenia Marques da Costa, Aline Renoult, Estelle Daudigeos-Dubus, Birgit Geoerger

Additional file 6: The uncropped Western Blots shown as part of Figure 5.

Figure 5C

<u>A204</u>

Figure 5C: p-ERK (42 & 44 kD)

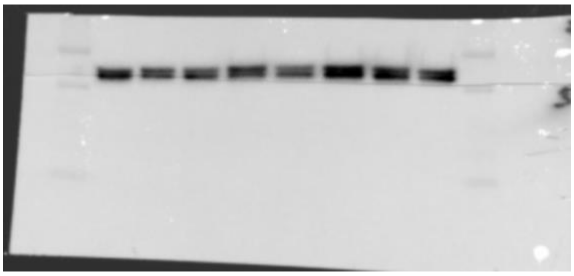

Figure 5C: ERK (42 & 44 kD, middle bands)

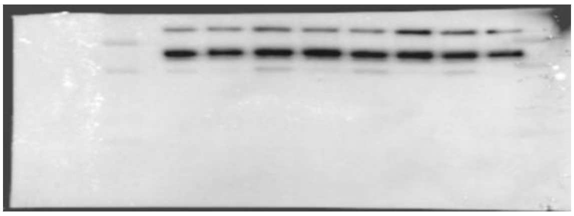

Figure 5C: p-AKT (60 kD)

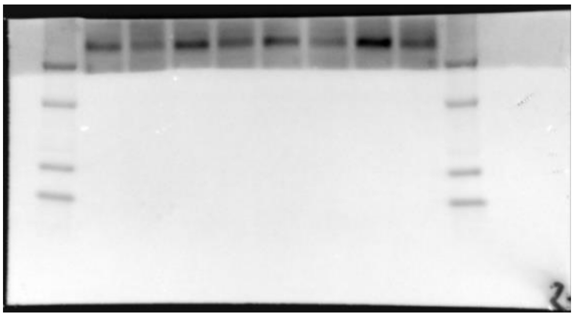

Figure 5C: AKT (60 kD, upper bands)

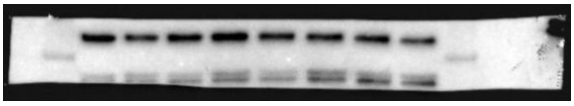

Figure 5C: PARP (110 kD)

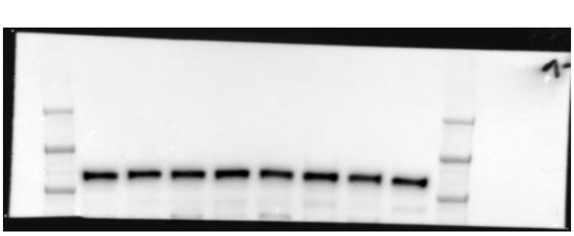

Figure 5C: cleaved PARP (89 kD, top bands)

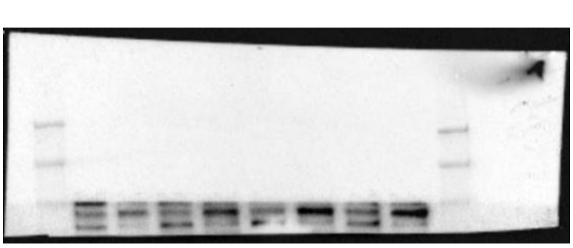

Figure 5C: β-Actin (45 kD)

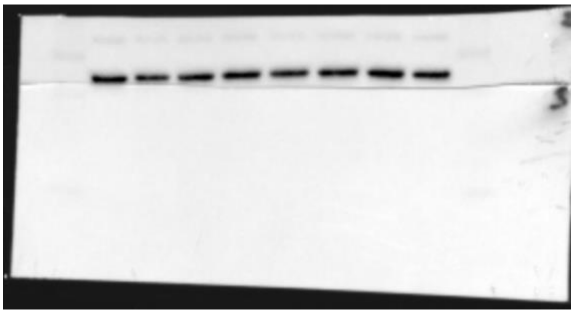

<u>VAESBJ</u>

Figure 5C: p-ERK (42 & 44 kD)

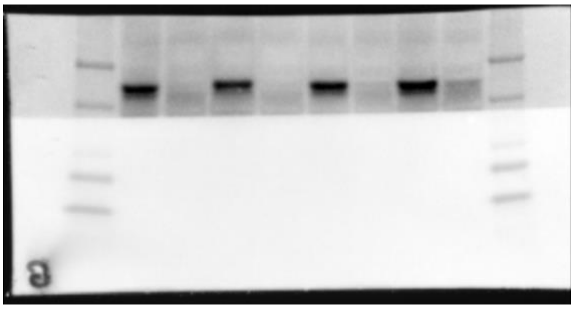

Figure 5C: ERK (42 & 44 kD)

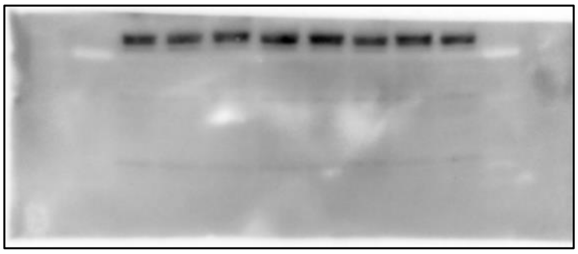

Figure 5C: p-AKT (60 kD)

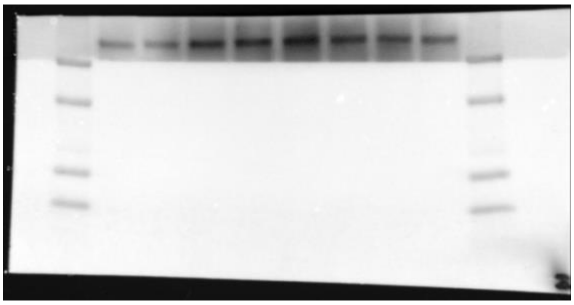

Figure 5C: AKT (60 kD)

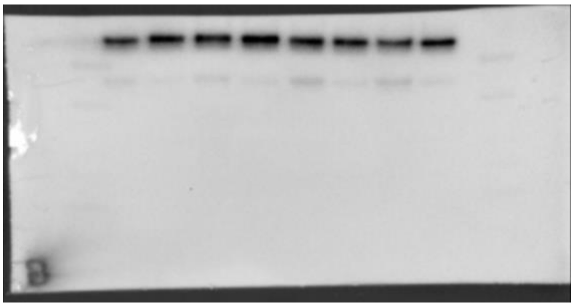

Figure 5C: PARP (110 kD, upper bands); cleaved PARP (89 kD, bottom bands)

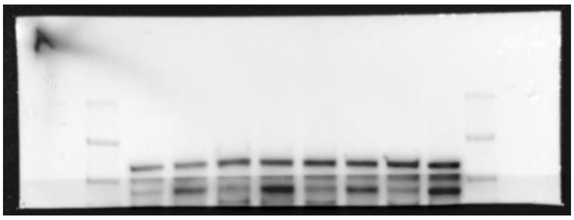

Figure 5C: β-Actin (45 kD)

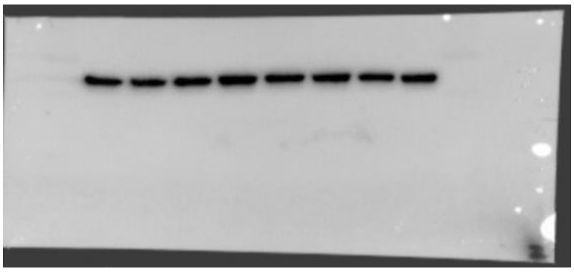

GRU1

Figure 5C: p-ERK (42 & 44 kD)

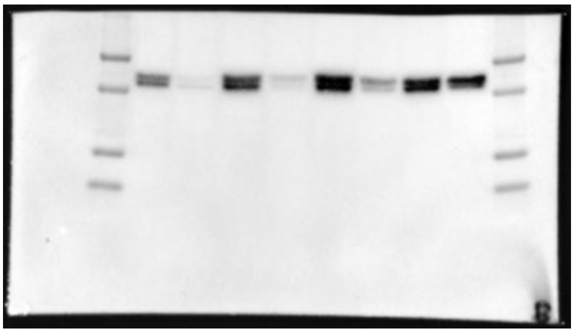

Figure 5C: ERK (42 & 44 kD)

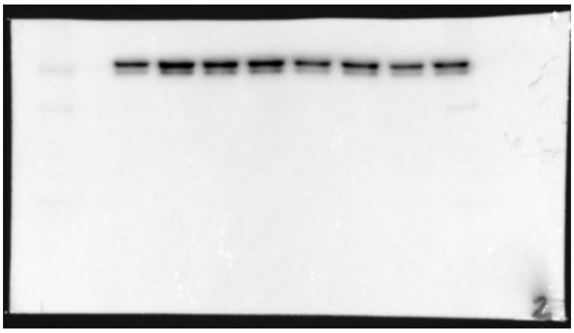

Figure 5C: p-AKT (60 kD)

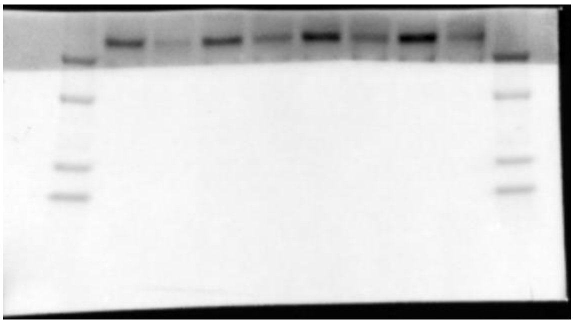

Figure 5C: AKT (60 kD)

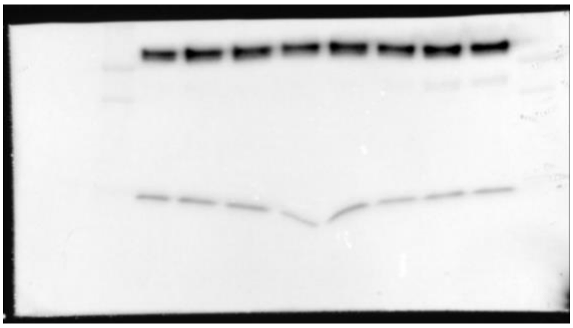

Figure 5C: PARP (110 kD)

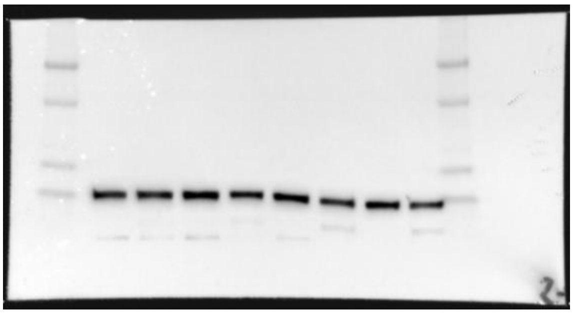

Figure 5C: cleaved PARP (89 kD)

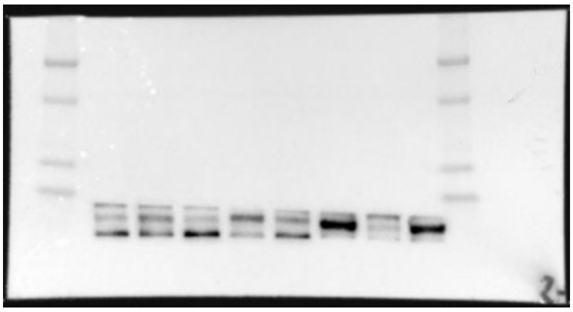

Figure 5C: β-Actin (45 kD)

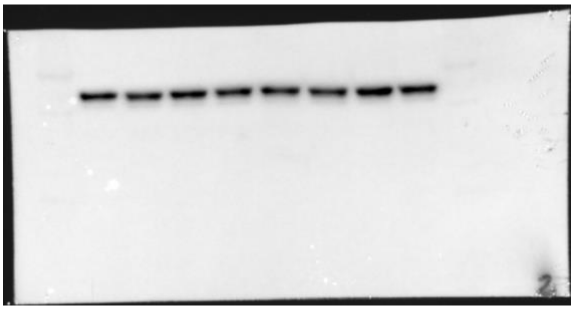

Supplement: Supplementary file 6 — Additional file 6. The uncropped Western Blots shown as part of Fig. 5. [file 12885_2021_8579_MOESM6_ESM.pdf]
