# Supplementary material for: Histone deacetylase inhibitor panobinostat induces antitumor activity in epithelioid sarcoma and rhabdoid tumor by growth factor receptor modulation
Source: BMC Cancer. 2021 Jul 20;21:833. doi: 10.1186/s12885-021-08579-w (PMC8290558; doi:10.1186/s12885-021-08579-w)
Supplement: Supplementary file 7 — Additional file 7. Details about treatment combinations shown in Fig. 5A. [file 12885_2021_8579_MOESM7_ESM.pdf]

Histone deacetylase inhibitor panobinostat induces antitumor activity in epithelioid sarcoma and rhabdoid tumor by growth factor receptor modulation

Anne Catherine Harttrampf, Maria Eugenia Marques da Costa, Aline Renoult, Estelle Daudigeos-Dubus, Birgit Geoerger

Additional file 7:

		Compound			
		Pazopanib	Erlotinib	EPZ011898-9	Panobinostat
Target	VEGFR1-3	x			
	PDGFRα, $-\beta$	x			
	FGFR1-3	x			
	FGFR_N549H	x			
	EGFR		x		
	KIT	x			
	FLT3	x			
	RET	x			
	TEK	x			
	wild-type EZH2			x	
	mutant EZH2			x	
	HDACs class I				x
	HDACs class II				x
	HDACs class IV				x

		Selected dose for treatment combination			
		Pazopanib	Erlotinib	EPZ011898-9	Panobinostat
Cell line	A204	IC_{50}			IC_{50}
	A204		10 µM		IC_{50}
	A204			10 µM	IC_{50}
	A204	IC_{50}		10 µM	
	VAESBJ	10 µM			IC_{50}
	VAESBJ		10 µM		IC_{50}
	VAESBJ				IC_{50}
	VAESBJ	10 µM		10 µM	
	GRU-1	10 µM			IC_{50}
	GRU-1		10 µM		IC_{50}
	GRU-1				IC_{50}
	GRU-1	10 µM		10 µM	
